# Gold nanoparticle distribution in advanced in vitro and ex vivo human placental barrier models

**DOI:** 10.1186/s12951-018-0406-6

**Published:** 2018-10-11

**Authors:** Leonie Aengenheister, Dörthe Dietrich, Amin Sadeghpour, Pius Manser, Liliane Diener, Adrian Wichser, Uwe Karst, Peter Wick, Tina Buerki-Thurnherr

**Affiliations:** 1 Empa, Particles-Biology Interactions, Swiss Federal Laboratories for Materials Science and Technology, Lerchenfeldstrasse 5, 9014 St. Gallen, Switzerland; 2 Institute of Inorganic & Analytical Chemistry, Westfälische Wilhelms-Universität Münster, Corrensstraße 28/30, 48149 Münster, Germany; 3 Empa, Center for X-ray Analytics, Swiss Federal Laboratories for Materials Science and Technology, Lerchenfeldstrasse 5, 9014 St. Gallen, Switzerland; 4 Empa, Laboratory for Advanced Analytical Technologies, Swiss Federal Laboratories for Materials Science and Technology, Ueberlandstrasse 129, 8600 Duebendorf, Switzerland

**Keywords:** Gold nanoparticle, Placental uptake and translocation, Ex vivo placenta perfusion, Placental in vitro co-culture model, Nanoparticle agglomeration

## Abstract

**Background:**

Gold nanoparticles (AuNPs) are promising candidates to design the next generation NP-based drug formulations specifically treating maternal, fetal or placental complications with reduced side effects. Profound knowledge on AuNP distribution and effects at the human placental barrier in dependence on the particle properties and surface modifications, however, is currently lacking. Moreover, the predictive value of human placental transfer models for NP translocation studies is not yet clearly understood, in particular with regards to differences between static and dynamic exposures. To understand if small (3–4 nm) AuNPs with different surface modifications (PEGylated versus carboxylated) are taken up and cross the human placental barrier, we performed translocation studies in a static human in vitro co-culture placenta model and the dynamic human ex vivo placental perfusion model. The samples were analysed using ICP-MS, laser ablation-ICP-MS and TEM analysis for sensitive, label-free detection of AuNPs.

**Results:**

After 24 h of exposure, both AuNP types crossed the human placental barrier in vitro, although in low amounts. Even though cellular uptake was higher for carboxylated AuNPs, translocation was slightly increased for PEGylated AuNPs. After 6 h of perfusion, only PEGylated AuNPs were observed in the fetal circulation and tissue accumulation was similar for both AuNP types. While PEGylated AuNPs were highly stable in the biological media and provided consistent results among the two placenta models, carboxylated AuNPs agglomerated and adhered to the perfusion device, resulting in different cellular doses under static and dynamic exposure conditions.

**Conclusions:**

Gold nanoparticles cross the human placental barrier in limited amounts and accumulate in placental tissue, depending on their size- and/or surface modification. However, it is challenging to identify the contribution of individual characteristics since they often affect colloidal particle stability, resulting in different biological interaction in particular under static versus dynamic conditions. This study highlights that human ex vivo and in vitro placenta models can provide valuable mechanistic insights on NP uptake and translocation if accounting for NP stability and non-specific interactions with the test system.

**Electronic supplementary material:**

The online version of this article (10.1186/s12951-018-0406-6) contains supplementary material, which is available to authorized users.

## Background

Gold nanoparticles (AuNPs) exhibit unique characteristics such as surface plasmon resonance, high stability in aqueous solutions, simple synthesis and modification of their surface properties, production with narrow size distributions and excellent compatibility with biological tissues [1, 2]. Therefore, they are widely explored in high technology applications such as organic photovoltaics, electronic conductors or catalysis as well as in the medical sector as potential tool in diagnostics, treatment and imaging [1–3].

NPs used as drug carriers demonstrate a promising opportunity for targeted treatment. Especially for pregnant women, it would be of great advantage to specifically treat only maternal, placental or fetal complications, avoiding potentially harmful side effects in the other compartments [4, 5]. However, before such a NP-based drug delivery strategy can be introduced to pregnant patients, its safety needs to be proven and the NPs need to be tailored in a specific way to steer their biodistribution and placental translocation in the expecting mother. Knowledge on how to control placental NP transfer via their physico-chemical properties or surface modifications is slowly emerging [4, 6, 7]. For instance, placental NP uptake and translocation has been shown to be dependent on particle size, surface modification and material composition [6]. Recently, specific targeting of NPs to the pregnant uterus was achieved to treat fetal growth restriction in mice [7]. Liposomes were loaded with a vasodilator and linked with a vascular targeting peptide, which enhanced accumulation of these NPs in the uterine vasculature and drug efficacy [7].

For AuNPs, studies investigating materno-fetal translocation and/or impact of the NPs on the placental barrier function and pregnancy are still rare and mostly done in pregnant rodents. However, since the placenta is probably the most species specific organ, it is important to confirm potential placental accumulation and transfer in human placenta models [8–10]. In humans, materno-fetal exchange occurs across four layers, namely the endothelial cells of the fetal capillary walls, the surrounding fetal stroma containing Hofbauer cells, a layer of mononucleated cytotrophoblasts (CT) and the multinucleated syncytiotrophoblast (ST) facing the maternal blood when the uteroplacental circulation is established after the first trimester of pregnancy. With ongoing gestation, the fetal capillaries multiply and migrate towards the thinning ST. At the same time, the CT cells flatten and partially disappear, resulting in a reduction of the barrier thickness to around 2–4 µm at term [11, 12].

In pregnant rats, Semmler-Behnke et al. [13] have shown that a substantial higher amount of 1.4 nm negatively charged, radio-labelled AuNPs were found in the placentas and in the amniotic fluid after intravenous injection compared to larger AuNPs (18 nm and 80 nm). However, only 1.4 and 18 nm AuNPs were found in the fetus, indicating either a transcellular entry of the AuNPs and/or a materno-fetal translocation via transtrophoblastic channels [13]. A study of Tsyganova et al. [14] confirmed a placental translocation of PEGylated AuNPs (5 and 30 nm) after intravenous injection in pregnant rats, but could not verify a size-dependent transfer. In mice, Yang et al. [15] demonstrated that fetal accumulation of AuNPs is dependent on their surface modification and the stage of pregnancy. In particular, substantially less uptake of 13 nm citrate-capped AuNPs in fetal tissues has been found compared to ferritin- or PEGylated-modified AuNPs in the same size. AuNP internalization was strongly reduced, when pregnant mice were exposed later than 11.5 days after successful mating (post embryonic day 11.5) [15]. 20 and 50 nm AuNPs intravenously injected on day 16 and 17 of gestation were found to accumulate in mouse placenta and maternal livers without detectable AuNP transfer to the fetal side [16]. No signs of toxicity were observed in placental, maternal and fetal tissue [16]. First studies on placental transfer of AuNPs using human models showed that 10 and 15 nm PEGylated-AuNPs did not cross the placental barrier after 6 h of ex vivo placental perfusion or a confluent layer of BeWo trophoblast cells after 48 h of exposure [17]. This is in apparent contradiction to most of the available studies in pregnant rodents, which observed placental transfer of small AuNPs (< approx. 20 nm) including PEGylated NPs [13–15]. Moreover, studies at other biological barriers such as the lung, also described a size-dependent translocation of AuNPs [18]. In general, only small percentages (below 2%) of the applied AuNPs have been shown to cross the alveolar barrier, however, significantly higher transfer rates were observed both in vitro and in vivo for very small AuNPs (< approx. 10 nm) [18–20]. Clearly, there is a need to better understand AuNP distribution at the human placental barrier, in particular, on the impact of particle size and surface modification on placental tissue uptake and materno-fetal transfer.

In this regard, we have previously found that 4 nm carboxylated AuNPs were taken up to a higher extent into the trophoblast layer of in vitro human placental co-culture microtissues (MTs) compared to 3 nm PEGylated AuNPs or larger (13–14 nm) particles of both modifications [21]. However, while 4 nm carboxylated AuNPs predominantly accumulated in the trophoblast layer, 3 nm PEGylated AuNPs penetrated into the fibroblastic core of the placental MTs raising the question whether these NPs could eventually overcome the human placental barrier [21]. Therefore, the aim of this study was to assess the uptake and translocation of small (3–4 nm) AuNPs with different surface modifications (PEGylate versus sodium carboxylate) at the human placental barrier. Since the bioavailable doses and uptake kinetics of NPs can be affected by the agglomeration/sedimentation behavior of NPs [22, 23] and the dynamic microenvironment [24], a further aim of the study was to investigate colloidal stability of the AuNPs in different biological media and to compare uptake and translocation in static and dynamic conditions. Hence, translocation studies were performed using a static human in vitro co-culture placenta model with tight monolayers of placental trophoblast cells (human choriocarcinoma cells; BeWo) and endothelial cells (human placental venous endothelial cells; HPEC-A2) cultivated on either side of a microporous membrane [25] as well as a dynamic human ex vivo placental perfusion model [26]. Uptake and translocation of AuNPs was investigated using laser ablation inductively coupled plasma mass spectrometry (LA-ICP-MS), sector-field-ICP-MS (SF-ICP-MS) and transmission electron microscopy (TEM). The agglomeration behavior of the AuNPs in the different culture media was monitored by small-angle X-ray scattering (SAXS).

## Methods

### Gold nanoparticles (AuNPs)

The AuNPs used in this study were provided within the EU FP7 Nanosolutions project and have been extensively characterized in our previous study (see Muoth et al. [21] for details on AuNP synthesis and characterization and Table 1 for a summary of the characteristics). Briefly, the AuNPs used in this study are 4 nm sodium carboxylate AuNPs (Au-4-COONa) and 3 nm PEGylate AuNPs (Au-3-PEG). Additional characterization was performed including zeta potential measurements of the AuNPs. 100 µg/mL AuNP suspensions were prepared in endothelial cell medium (EM) or perfusion medium (PM) (stock suspensions were sonicated for 5 min before addition) and zeta potential was immediately determined at 25 °C using a Zetasizer device (Nanoseries, Nano-ZS90, Malvern, Worcestershire, UK). Suspensions were measured in disposable folded capillary cells (DTS 1070; Malvern Instruments Ltd., Worcestershire, UK) with 2 min equilibration time and five consecutive measurements (with at least 10 and maximum of 100 runs).

**Table 1:** AuNP characteristics

	Au-3-PEG NPs	Au-4-COONa NPs
TEM: primary particle size (nm)^a^	3.5 ± 1.2	4.5 ± 1.5
Zeta potential in H_2_O (mV)^a^	− 16.5 ± 3	− 28.8 ± 0.4
Zeta potential in EM (mV)	− 9.4 ± 1.4	− 8.5 ± 0.8
Zeta potential in PM (mV)	− 6.8 ± 0.7	− 9.1 ± 0.4
Surface functionalization	SH-PEG_(550)_CH_3_	SH-(CH_2_)_10_-COONa
ICP-OES: Au content (%)^a^	76.98	38.38

Data shown as mean ± SD

*TEM* transmission electron microscopy, *ICP-OES* inductively coupled plasma optical emission spectroscopy, *SD* standard deviation

^a^According to [21]

### Small angle X-ray scattering (SAXS)

The agglomeration of NPs in nanoscale has been studied by SAXS experiments. A Nanostar SAXS device (Brucker AXS GMBH, Karlsruhe, Germany) equipped with a microfocus X-ray source (Cu Kα radiation; 0.154 nm wavelength) and MONTEL optics providing a point-focused beam diameter of about 300 µm was used. A VÅNTEC-2000, Xe-based gaseous avalanche detector, capable of photon counting with 0.5 s temporal resolution was used to record the 2-dimensional scattering patterns. Suspensions of 50 µg/mL Au-4-COONa and 25 µg/mL Au-3-PEG in EM or ultrapure water (0 h, 6 h, 24 h) as well as 25 µg/mL suspensions of both NPs in PM (0 h, 6 h) were incubated at 37 °C/5% CO_2_ in Eppendorf tubes or 24 well plates under static conditions. Pictures and light microscopic images (Primovert, Zeiss, Feldbach, Switzerland) of the suspensions were taken for a qualitative assessment of NP sedimentation, prior to X-ray studies. For SAXS measurements, suspensions (excluding the pellet) were transferred into quartz capillaries of 1.5 mm in outer diameter (Hilgenberg GmbH, Malsfeld, Germany). The capillaries were vacuum tightened using wax sealing. The scattering profiles of empty and water filled capillaries were obtained under the same conditions. A semi-transparent beam stop enabled normalization of the curves and subtraction of background noise. The instrument was operated at sample to detector distances of about 107 cm providing access to the minimum scattering vector modulus [*q* = (4π/λ) sin(θ), where 2θ is the scattering angle] of 0.06 nm^−1^. The 2-D scattering patterns for each sample were recorded 6 times, each over an acquisition time of 20 min and then averaged. Subsequently, the patterns were azimuthally integrated to achieve the 1-D scattering profiles, represented by the scattering intensity as a function of scattering vector modulus. All SAXS experiments were performed at room temperature (RT) after incubation in different media at 37 °C. The data analysis has been performed by the use of generalized indirect Fourier transformation [27, 28] enabling us to achieve the pair-distance distribution function (PDDF). From these data, the average radius of gyration as a quantitative measure of particle size has been identified.

### Dynamic light scattering (DLS)

The hydrodynamic diameter of the NPs was measured in 100 µg/mL AuNP suspensions in EM, PM and ultrapure H_2_O at 37 °C for 0, 6 and 24 h, respectively. Just before measurement, the Zetasizer device (Nanoseries, Nano-ZS90, Malvern, Worcestershire, UK) equipped with the standard 633 nm laser was set to 37 °C and the pre-heated suspension was added to the cuvette (UV-transparent disposable; Sarstedt Ag + Co, Nümbrecht, Germany) followed by the addition of the required amounts of NPs (stock suspensions were sonicated for 5 min before addition) and short mixing. The experiment was started immediately and the first measurement was considered as timepoint “0 h”. The measurement duration was set to automatic mode without delays and the measurement angle was 90°. The actual temperature of the NP suspension in the cuvette was recorded beforehand over 3 h using a temperature data logger (MSR 145 B4, MSR Electronics GmbH, Seuzach, Switzerland) and was determined to lie between 33.6 and 35.0 °C instead of 37.0 °C.

### Cell culture and in vitro barrier formation

HPEC-A2 cells (SV40-transformed microvascular human placental venous endothelial cells) and the human placental choriocarcinoma cell line BeWo b30 were provided by Prof. G. Desoye [Department of Obstetrics and Gynecology, Medical University Graz, Graz, Austria (with permission from Prof. P. Friedl, Institute of Biochemistry, Technical University Darmstadt, Darmstadt, Germany)] and Prof. Dr. Ursula Graf-Hausner [Zurich University of Applied Science (with permission from Dr. Alan L. Schwartz, Washington University School of Medicine, MO, USA)], respectively. General cell cultivation conditions of HPEC-A2 cells and BeWo cells as well as monolayer and co-culture formation were done as described previously [25]. Briefly, polycarbonate Transwell^®^ inserts (pore size 3.0 µm, growth area 1.12 cm^2^, apical volume 0.5 mL, basolateral volume 1.5 mL; Corning^®^, Sigma-Aldrich, Buchs, Switzerland) were pre-coated with 50 µg/mL human placental collagen IV (Sigma-Aldrich, Buchs, Switzerland) for 1 h at 37 °C/5% CO_2_. Monolayer formation was obtained by seeding either 1.5 × 10^5^ BeWo cells on the apical side or 1 × 10^5^ HPECs on the basolateral side of the membrane. For the co-culture, HPECs were cultivated on the basolateral side for 2 h followed by seeding BeWo cells on the apical side. Cells were cultivated for 3 days at 37 °C/5% CO_2_ in endothelial cell growth medium MV supplemented with 1% penicillin/streptomycin (pen/strep, Gibco, Luzern, Switzerland) and SupplementMix according to the manufacturer’s guide (PromoCell, Heidelberg, Germany; further referred to as EM) under static conditions (medium change after 48 h).

### In vitro translocation of AuNPs

Co-cultures as well as monolayers (HPECs or BeWo cells on the basolateral or apical side of the insert, respectively) were cultivated for 3 days. To determine placental translocation in vitro, fresh EM was given to the basolateral chamber and 50 µg/mL of Au-4-COONa (19.2 µg/mL Au) or 25 µg/mL Au-3-PEG NPs (19.2 µg/mL Au) were applied apically. Cultures were then further incubated for 24 h at 37 °C/5% CO_2_ under static conditions. The transepithelial electrical resistance (TEER) was measured before and after the treatment to ensure barrier formation before the experiment and to determine a potential influence of the AuNPs on barrier integrity after 24 h. At the end of the treatment, apical and basolateral supernatants were collected and membranes were kept in fresh pre-warmed EM for TEER measurement. Membranes were cut off from the holder with a scalpel and all samples were stored for further ICP-MS analysis at 4 °C. Samples were also taken from the AuNP working suspensions and EM of each experiment to determine Au concentration at the beginning of the experiment (0 h).

### Ex vivo translocation of AuNPs

Placentas from uncomplicated term pregnancies were obtained after caesarean section at the Kantonsspital and the Hirslanden Klinik Stephanshorn in St. Gallen (Switzerland). The project was approved by the local ethics committee, performed in accordance with the principles of the Declaration of Helsinki and written informed consent was given by the expecting mothers prior to delivery. The ex vivo placental perfusions were performed in a dually perfused, closed system as described before [26]. The perfusion medium (further referred to as PM) consisted of M199 tissue culture medium, which was diluted with Earl’s buffer (1:2) and further supplemented with glucose (1 g/L), bovine serum albumin (BSA; 10 g/L), dextran 40 (10 g/L), sodium heparin (2500 IU/L), amoxicillin (250 mg/L) and sodium bicarbonate (2.2 g/L; medium and all supplements were obtained from Sigma-Aldrich, Buchs, Switzerland). A perfusion was considered successful if: (1) the pre-perfusion of the tissue showed no leakage, (2) the leakage (fetal to maternal) was less than 4 mL/h during the translocation experiment and (3) the pH stayed constant during the experiment (7.2–7.4). After establishing the system, Au-4-COONa and Au-3-PEG were added to the maternal chamber to obtain a final concentration of 25 µg/mL. Maternal and fetal samples were taken at 0, 0.25, 0.5, 1, 2, 3, 4, 5 and 6 h of perfusion and analyzed immediately for pH levels using a blood gas analyzer (Epocal Inc., Ottawa, Canada). Afterwards, supernatants were stored at − 20 °C for further ICP-MS analysis. In addition, placental tissue samples were taken before and after perfusion and stored at − 20 °C for ICP-MS analysis as well as in 4% paraformaldehyde (PFA; Sigma-Aldrich, Buchs, Switzerland) at RT for LA-ICP-MS analysis.

### Transmission electron microscopy (TEM)

TEM was conducted to investigate AuNP internalization in BeWo cells. Therefore, cells were cultivated on inserts for 3 days as described above and treated with 50 µg/mL Au-4-COONa or 25 µg/mL Au-3-PEG for 24 h at 37 °C and 5% CO_2_. Cells were detached from 11 inserts per condition (without NPs, Au-3-PEG, Au-4-COONa) using 0.5% Trypsin–EDTA, pelleted by centrifugation (200 g, 5 min) and sucked up into a capillary tube (Leica-Microsystems). Cells enriched in the capillaries were immediately fixed in 3% glutaraldehyde in 0.1 M sodium cacodylate buffer and washed in 0.2 M sodium cacodylate buffer. After a post-fixation step in 2% osmium tetroxide in 0.1 M sodium cacodylate buffer, samples were dehydrated through a graded ethanol series followed by acetone and finally embedded in Epon resin (Sigma-Aldrich, Buchs, Switzerland). Ultrathin sections were contrasted with 2% uranyl acetate and lead citrate (Reynolds 1963) before imaged in a Zeiss EM 900 (Carl Zeiss Microscopy GmbH, Germany) at 80 kV.

### Sector field-inductively coupled plasma-mass spectrometry (SF-ICP-MS)

To determine the Au content of the ex vivo samples, 1 g placental tissue (taken before and after each perfusion) was homogenized in 3 mL perfusion medium using a TissueRuptor (Qiagen, Hilden, Germany). Samples of the maternal and fetal solution (each 250 µL) and homogenized tissue (~ 250 µL corresponding to 83.3 mg tissue) were digested in 0.6 mL concentrated nitric acid and 1.8 mL hydrochloric acid (both NORMATOM, VWR Chemicals, Vienna, Austria) using a microwave (turboWAVE Inert, MLS GmbH, Leutkirch, Germany). In vitro samples (50–200 µL of supernatants or membranes) were digested in HCL and nitric acid (1:2 ratio to sample) for 3 days at RT. Processed samples were further diluted using ultrapure water. Au content was determined by SF-ICP-MS (Element 2, Thermo Finnigan, Bremen, Germany) with external calibration ranging from 0 to 50 µg/L. Rhenium was added to the ex vivo samples as internal standard. The isotope ^197^Au determined in low resolution was used for quantification. The minimum detection limits of ^197^Au were 0.041 µg/L (ex vivo) and 0.016 µg/L (in vitro) (3*SD). Therefore, approx. 0.0052% (Au-3-PEG NPs) and 0.0085% (Au-4-COONa NPs) of the initial dose (ID) could have been detected ex vivo in the fetal chamber and 0.014% of the ID in vitro on the basolateral side after 24 h.

### Laser ablation-inductively coupled plasma-mass spectrometry (LA-ICP-MS)

Elemental bioimaging and quantification of Au in ex vivo placental tissue and in vitro monolayers was conducted by LA-ICP-MS analysis using 15 µm paraffin sections of placental tissue or membranes from translocation studies stained with hematoxylin/eosin (H&E). The laser ablation system LSX-213 G2+ (Teledyne CETAC Technologies, Omaha, NE, USA) with a frequency-quintupled Nd:YAG laser (213 nm) was coupled to a quadrupole-based ICP-MS (iCapQc, Thermo Fisher Scientific, Bremen, Germany). To achieve optimal spatial resolution, the smallest possible laser spot size of 4 µm was selected. For analysis of ex vivo samples, a scan speed of 12 µm/s and line by line ablation with 0 µm space between the lines was applied. For smaller in vitro samples, a scan speed of 6 µm/s was selected and line by line ablation was conducted with overlapping lines (space between the lines of − 2 µm). This oversampling approach resulted in a virtual spot size of 2 µm under quantitative ablation conditions. Both tissue samples were analyzed with a laser shot frequency of 20 Hz and the laser energy was adjusted to assure quantitative ablation of the samples (9 J cm^−2^). The ablation chamber was flushed with 800 mL/min helium carrier gas flow that was mixed with 1.02 L/min argon before entering the plasma. The ICP-MS was equipped with platinum sampler and skimmer cones and a quartz injector pipe with 3.5 mm inner diameter for sample introduction. RF power was set to 1550 W and other parameters were tuned daily to maximum intensities for laser ablation coupling. For analysis of ex vivo samples, the isotopes ^27^Al, ^79^Br (dwell time 55 ms each), ^31^P and ^197^Au (110 ms each) were monitored throughout the analysis. For in vitro samples, isotopes ^79^Br (55 ms) and ^197^Au (110 ms) were detected. Aluminum and bromine were detected as a result of the hematoxylin and eosin staining of the sample to obtain an improved allocation of tissue features in the microscopic and mass spectrometric images, respectively. Due to possible polyatomic interferences on the isotopes ^27^Al, ^31^P and ^79^Br, ICP-MS analysis was conducted in kinetic energy discrimination (KED) mode with helium as cell gas at a flow rate of 4.2 mL/min.

Transient signals were converted into color-coded elemental distribution maps using *MassImager* software, written by Robin Schmid (University of Münster, Karst research group). To convert detected signal intensities into concentrations, quantification was carried out by external calibration with matrix-matched standards based on 10% gelatin (*w*/*v*). Gelatin (100 mg, Grüssing GmbH Analytica, Filsum, Germany) was spiked with 900 µL gold suspensions (0.11–111 µg/g gold) that were prepared by dilution of a stock solution of 1000 µg/g gold (CentriPur Merck, Darmstadt, Germany). Mixtures were homogenized at 40 °C with frequent shaking. For calibration, eight standards with concentrations between 0.1 µg/g and 100 µg/g were prepared. Approx. 80 µL of the mixtures were pipetted onto a sample holder, cooled to − 23 °C and sectioned to 15 µm thicknesses using a cryomicrotome (CryoStar NX70, Thermo Scientific, Waltham, MA, USA). Standard sections were analyzed by LA-ICP-MS using the same experimental parameters as applied for the tissue sections on the same day. Eleven lines per standard concentration were ablated with durations of 28 s each. While the first line was discarded, the signals of the following ten lines were used for quantification. For evaluation of total standard concentrations, 200 mg of each standard were dissolved in 300 µL *aqua regia.* Solutions were mixed with equal amounts of arsenic in 6% HNO_3_ as internal standard (diluted from arsenic ICP standard 1000 µg/g, Merck, Darmstadt, Germany). Aliquots of 5 µL were dried onto quartz glass discs (Bruker nano GmbH, Berlin, Germany) and analyzed by total reflection x-ray fluorescence (S2 Picofox, Bruker nano GmbH). The X-ray tube was equipped with a molybdenum anode working at an anode current of 750 μA and voltage of 50 kV. Detection was carried out using an energy-dispersive, Peltier-cooled silicon drift-detector (SDD, XFlash). Signal integration time was set to 1000 s. For data evaluation, the software Spectra Picofox Version 7.2.5.0 (Bruker AXS) was used.

### Statistics

The experimental data is presented as mean ± standard deviation (SD). To identify statistical difference in Au distributions in vitro each compartment was compared to the respective control (without cells) or to each other (e.g. BeWo vs. HPEC) using an unpaired student’s t-test. Statistical significance was obtained when P < 0.05 (*).

## Results

### Characterization of the AuNPs

The Au-3-PEG and Au-4-COONa NPs have already been characterized in a previous study, and results are summarized in Table 1 [21]. In addition, zeta potentials of Au-3-PEG and Au-4-COONa were measured in EM (used for in vitro translocation studies) and PM (used for ex vivo translocation studies) and were slightly negative, similar to values determined from EM and PM only (data not shown).

**Fig. 1:**
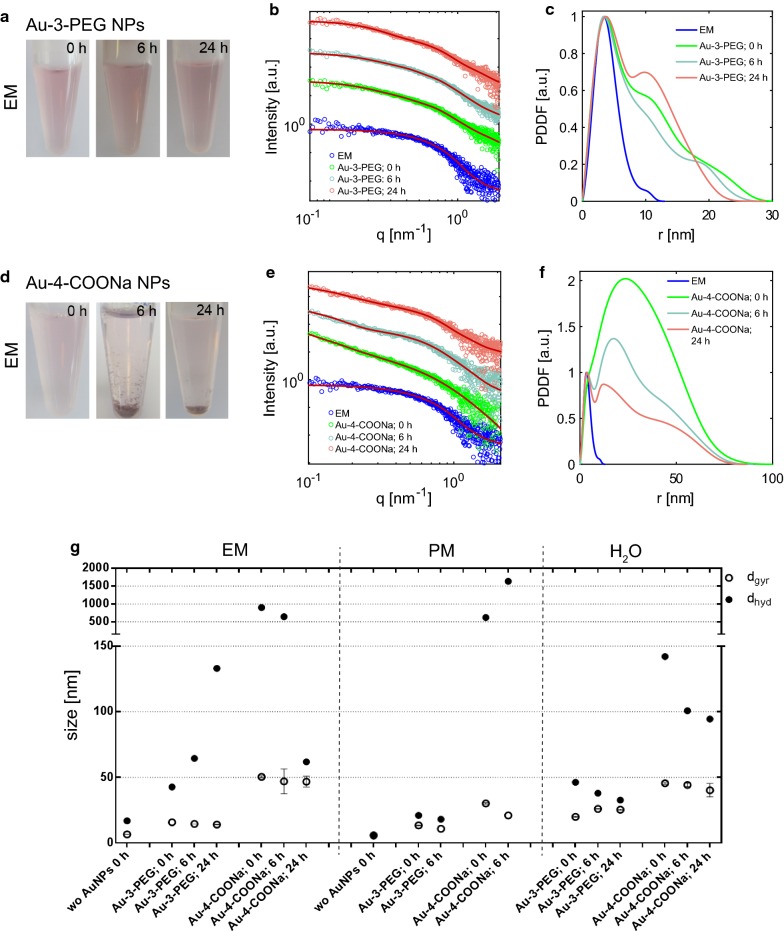
Colloidal stability of Au-3-PEG (**a**–**c**) and Au-4-COONa (**d**–**f**) NPs in EM. **a**, **d** Au-3-PEG and Au-4-COONa NP suspensions in EM (19.2 μg**/**mL Au each) after 0, 6 and 24 h of static incubation (37 °C/5% CO_2_) in the absence of cells. **b**, **e** Scattering intensity as function of scattering vector q and **c**, **f** pair distance distribution function (PDDF) of the PEGylated and carboxylated AuNPs in EM. **g** Diameter of gyration (d_gyr_; mean ± error) calculated from SAXS data and hydrodynamic diameter from DLS analysis (d_hyd_; Z-Average)

Colloidal stability of the AuNP suspensions in the different media was investigated by visual observation, SAXS and DLS. Suspensions with experimental concentrations of the AuNPs (25 µg/mL of Au-3-PEG and 50 µg/mL Au-4-COOH NPs in EM or H_2_O; 25 µg/mL of both AuNP types in PM) were incubated for 0 h, 6 h (maximum for ex vivo perfusion experiments) and 24 h (maximum for in vitro translocation studies) at 37 °C/5% CO_2_ under static conditions. No pellet formation was apparent for Au-3-PEG NPs suspensions in EM, PM or H_2_O (Fig. 1a, Additional file 1: Fig. S1a, g). In contrast, a pellet was observed for Au-4-COONa NPs dispersed in EM and PM (Fig. 1d, Additional file 1: Fig. S1d) but not in H_2_O (Additional file 1: Fig. S1j). Light microscopic images of EM and PM suspensions confirmed the formation of a NP precipitate at the bottom of the well plates for Au-4-COONa NPs while no particles were detected for Au-3-PEG NP dispersions even after 24 h of incubation (Additional file 1: Fig. S3). To determine particle/agglomerate sizes in the soluble NP fraction, SAXS measurements were performed from the supernatant without disturbing the pellet. The precipitated fraction was not analyzed since the maximum particle size that can be detected by SAXS is limited to approximately 100 nm. Therefore, DLS was used as a complementary method to also detect larger NP agglomerates. SAXS analysis revealed that Au-3-PEG NPs dispersed in EM were relatively stable, and only a minor increase in the smaller particle fraction (r ~ 10–15 nm) and concomitant decrease in the larger particle fraction (r ~ 20–30 nm) was observed after prolonged incubation for 24 h (Fig. 1c), indicating a slight dissociation process. The diameter of gyration (d_gyr_) slightly decreased over time from 15.7 nm at 0 h to 14.1 nm at 24 h, whereas the hydrodynamic diameter (d_hyd_) obtained with DLS increased from 42.8 nm at 0 h to 133 nm at 24 h of incubation at 37 °C (Fig. 1g, Additional file 1: Fig. S2). For Au-4-COONa suspensions in EM, a strong decrease in PDDF was detected over 24 h of incubation, which is indicative of particle sedimentation and lower AuNP concentrations (Fig. 1e, f). While the d_gyr_ of the suspended particle fraction remained similar (50.4 nm at 0 h vs. 46.8 nm at 24 h), a major decrease was observed in the d_hyd_ (902.1 nm at 0 h, 642.5 nm at 6 h and 61.9 nm at 24 h) (Fig. 1g, Additional file 1: Fig. S2). This was accompanied by a substantial decrease of the average scattered intensity of light from 1707.4 kcps at 0 h to 87.3 kcps at 6 h and to 57.3 kcps at 24 h (derived count rates) measured by DLS over 24 h. Taken together, this indicates that Au-4-COONa NPs quickly agglomerate in EM, with bigger particles leaving the detection window due to sedimentation. In PM, similar colloidal stability was observed as in EM (Additional file 1: Fig. S1). Au-3-PEG NPs showed a minor decrease of d_gyr_ from 13.4 nm at 0 h to 10.8 nm at 6 h and of d_hyd_ from 21 nm at 0 h to 18.1 nm at 6 h, respectively (Fig. 1g, Additional file 1: Fig. S2). For Au-4-COONa NPs in PM, the amount of particles was reduced after 6 h (Additional file 1: Fig. S1e, f) and the d_gyr_ decreased from 30.2 nm at 0 h to 21.0 nm at 6 h (Fig. 1g). The d_hyd_ of Au-4-COONa NPs in PM was 621.3 nm at 0 h (1745.9 kcps) and 1634 nm after 6 h (1186.5 kcps) of incubation, revealing particle agglomeration (Fig. 1g, Additional file 1: Fig. S2). In contrast to Au-4-COONa dispersions in EM, an increase in d_hyd_ was observed suggesting that in PM these particles agglomerate more slowly and therefore do not readily leave the detection window in DLS. In water, both AuNPs appeared to stay in suspension (Additional file 1: Figs. S1, S2). Au-3-PEG NPs demonstrated a slight increase of d_gyr_, (19.9 nm at 0 h vs. 25.4 nm at 24 h) but a small decrease of d_hyd_ over time (46.28 nm at 0 h vs. 32.66 nm at 24 h), respectively (Fig. 1g). In contrast to EM or PM, Au-4-COONa NPs displayed a high colloidal stability in water, as evidenced by highly stable signal intensities during SAXS measurements (Additional file 1: Fig. S1k, l) and a minimal decrease in d_gyr_ (45.6 nm at 0 h vs. 40.2 nm at 24 h) and d_hyd_ (142 at 0 h vs. 94.4 nm at 24 h; Fig. 1g).

### Determination of AuNP distribution using a static in vitro co-culture placental barrier model

To ensure the absence of acute toxicity, cell viability was assessed prior to the translocation studies. Exposure of BeWo cells for up to 48 h with AuNP concentrations ranging from 0 to 50 µg/mL did not affect their viability (Additional file 1: Fig. S4).

**Fig. 2:**
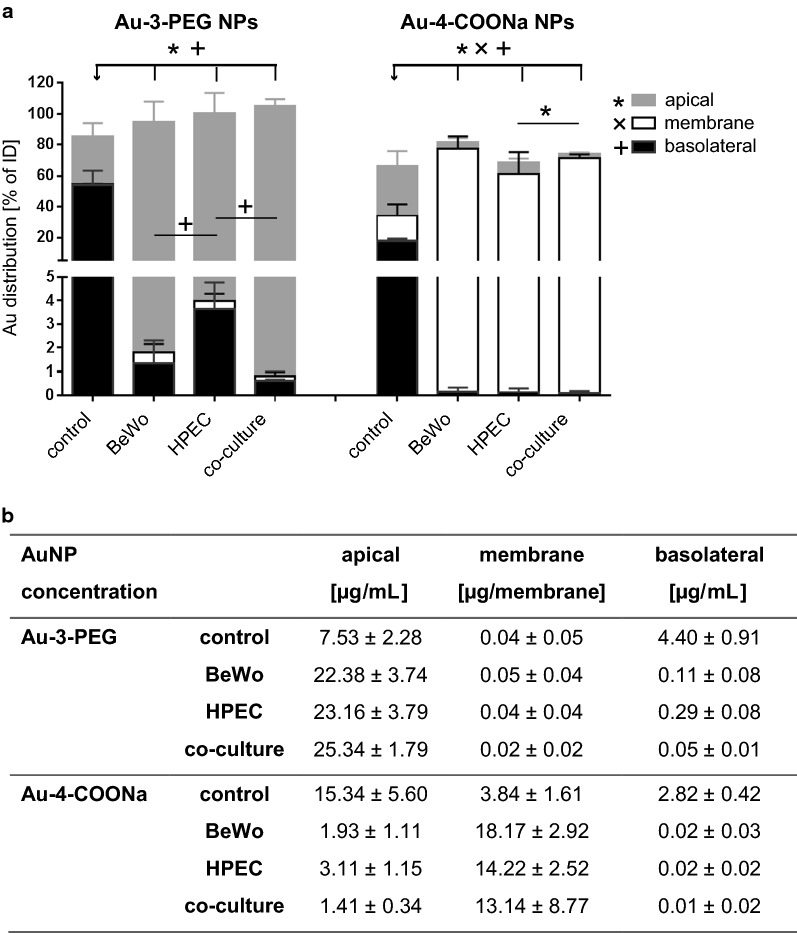
Au distribution in the static in vitro placental barrier model after exposure to Au-3-PEG and Au-4-COONa NPs for 24 h. Control membranes, monocultures or co-cultures were exposed to 19.2 μg**/**mL Au from Au-3-PEG and Au-4-COONa NPs for 24 h and Au content was determined in apical and basolateral supernatants as well as in the membrane fraction using SF-ICP-MS. Distribution of the Au amount as % of the initial dose (ID; **a**) and absolute concentrations of the AuNPs (**b**) in the apical and basolateral chamber and in the membrane fraction, respectively. The latter were calculated from the Au concentrations measured by SF-ICP-MS using the relative Au content of the NPs determined with ICP-OES (see Table 1). Data represent the mean ± SD of 3–4 biologically independent experiments with one technical replicate each. P-value below 0.05 are considered statistically significant (*, x and + are related to apical, membrane and basolateral values)

In vitro placental translocation studies were performed across tight layers of trophoblast cells (BeWo) only, placental microvasculare endothelial cells (HPEC) only or the co-cultures (BeWo/HPEC) in static conditions. To allow for direct comparison of the NPs containing different amounts of Au, cell cultures were exposed to the same Au content, namely 19.2 µg Au/mL (corresponding to 25 µg/mL Au-3-PEG and 50 µg/mL Au-4-COONa NPs). ICP-MS measurements revealed that around 54.2% and 17.8% of Au from Au-3-PEG and Au-4-COONa NPs were able to cross the cell-free microporous membrane (Fig. 2), corresponding to concentrations of 4.40 µg/mL Au-3-PEG NPs and 2.82 µg/mL Au-4-COONa NPs. Of note, an equilibrium of the AuNP concentrations would be reached when three quarters of the added NP amount would reach the basolateral chamber [which is 75% of the initial dose (ID)], since apical and basolateral volumes in the bi-chamber system are 0.5 and 1.5 mL, respectively. Au-3-PEG and Au-4-COONa NPs were highly retained by the HPEC and BeWo monolayer as well as the co-culture even after 24 h of exposure. Only 1.3, 3.6 and 0.6% of the ID of Au-3-PEG NPs and 0.2, 0.1 and 0.1% of the ID of the Au-4-COONa NPs were detected on the basolateral side of BeWo and HPEC monolayers or co-cultures, respectively. Significant differences in the retention capability of the distinct cell layers were only observed for the HPEC layer, which was more permeable to Au-3-PEG NPs as compared to the BeWo or co-culture layers. Only approximately 80% of the ID of the carboxylated AuNPs was found by ICP-MS measurements, indicating a potential adsorption to the insert holder and/or loss of precipitated particles during washing of the membrane (addition of fresh medium for TEER measurements).

**Fig. 3:**
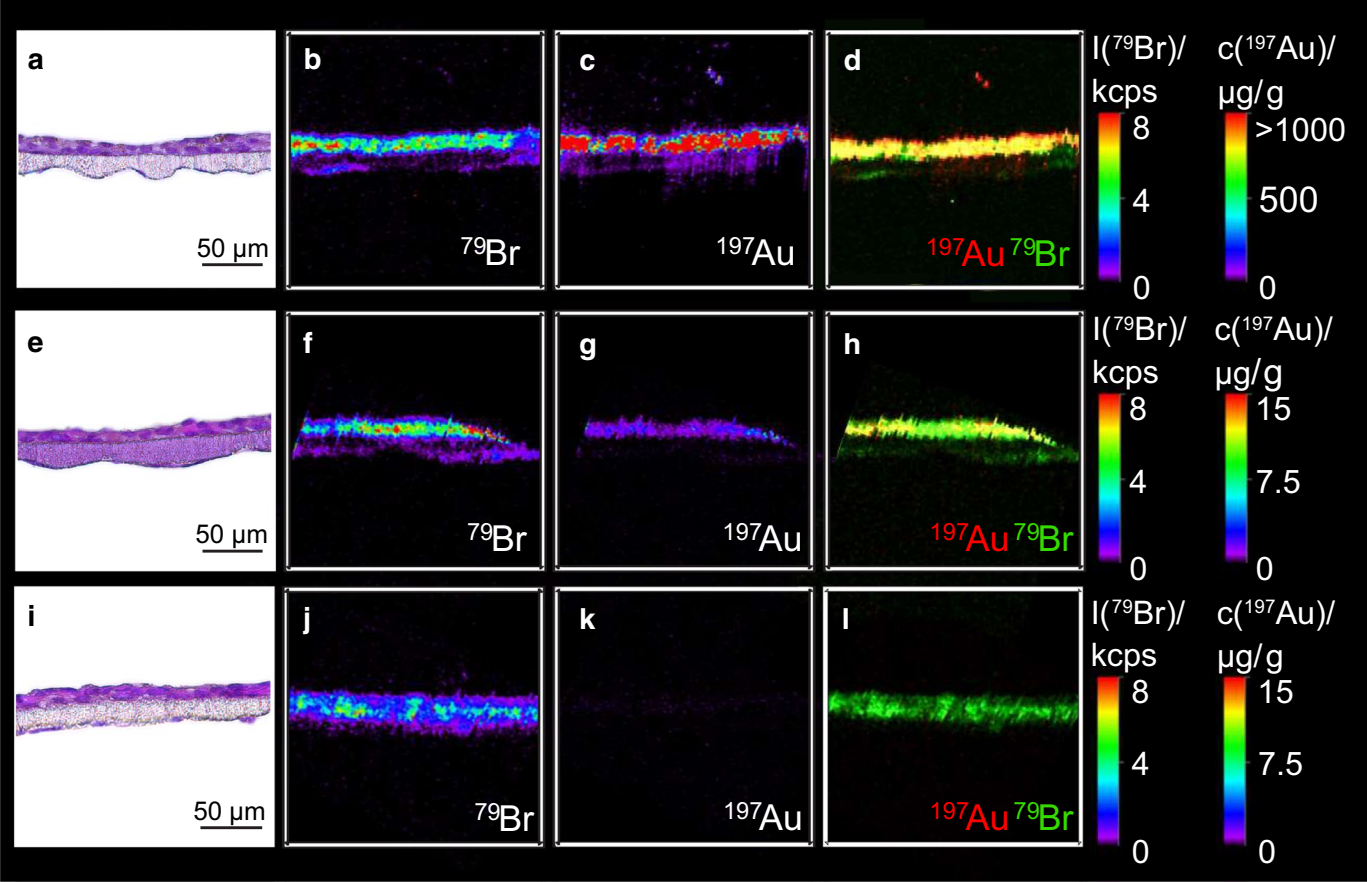
Distribution of Au-4-COONa and Au-3-PEG NPs in the in vitro placental barrier model after 24 h. Co-cultures were exposed to 19.2 μg/mL Au from Au-4-COONa (**a**–**d**) and Au-3-PEG (**e**–**h**) NPs for 24 h under static conditions. A co-culture without NP treatment was used as negative control. H&E staining of 5 µm thick sections of the co-culture enables allocation of Au signals (**a**, **e**, **i**; adjacent sections to those for LA-ICP-MS). Elemental distribution of ^79^Br to visualize cell structure (**b**, **f**, **j**). ^197^Au was measured to localize Au (**c**, **g**, **k**). Overlay of ^79^Br and ^197^Au to co-localize Au signals in cells (**d**, **h**, **l**). Elemental distribution map of ^79^Br and ^197^Au is represented in signal intensities and Au concentrations (black: minimum, red: maximum), respectively. Images were obtained from one independent experiment

When analyzing the Au content in the membrane fraction, only very low amounts of Au-3-PEG NPs were detected (0.5, 0.3, 0.2% of the ID) in BeWo, HPEC and co-cultures, respectively. In contrast, almost all Au-4-COONa NPs were found to be internalized by the cells and/or adsorbed on the cell surface in BeWo monolayers (77.4% of ID), HPEC monolayers (61.4% of ID) and co-cultures (71.2% of ID). Au content in cell free membranes were 0.3% of ID for Au-3-PEG NPs and 16.5% of ID for Au-4-COONa NPs. Overall, Au-3-PEG and Au-4-COONa NPs demonstrate a substantially different in vitro distribution behavior, which was confirmed by LA-ICP-MS (Fig. 3).

**Fig. 4:**
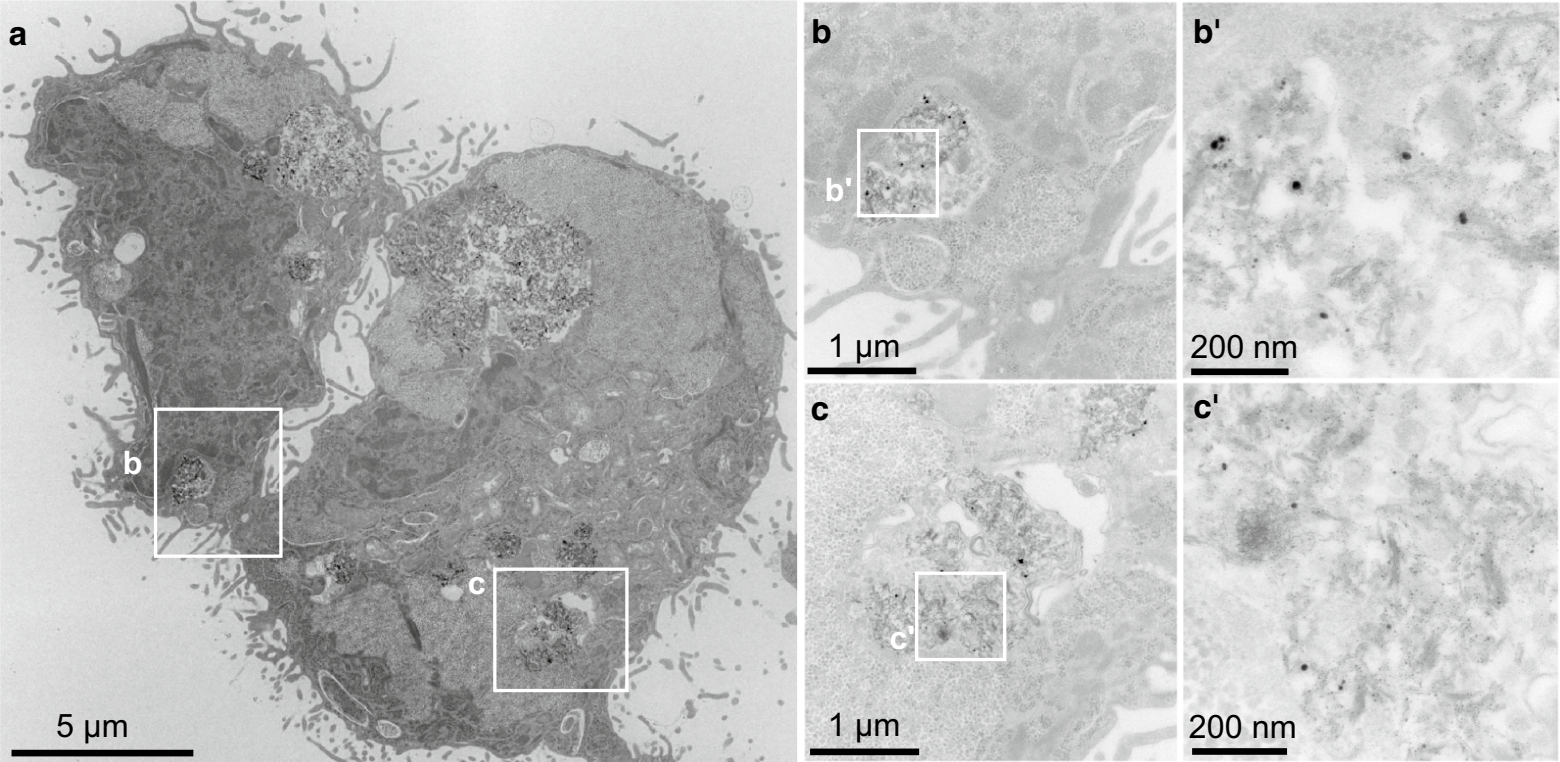
Uptake of Au-4-COONa NPs in BeWo cells after 24 h. **a** TEM micrographs showing an overview of two BeWo cells. Two different locations with internalized AuNPs (white squares **b** and **c**) are presented at higher magnifications (**b**, **b'** and **c**, **c'**)

Mean Au concentrations of 1370 µg/g and 0.6 µg/g were detected in the co-culture layers after Au-4-COONa and Au-3-PEG NP treatment for 24 h, respectively. While most AuNPs were detected evenly distributed in the BeWo cell layer, only low signals were measured in the membrane and the HPEC layer. TEM micrographs further verified the uptake of Au-4-COONa NPs in BeWo cells, and particles were mainly located in membrane-bound vesicles in the form of small agglomerates (Fig. 4). In contrast, no NPs were observed in BeWo cells exposed to Au-3-PEG NPs for 24 h (Additional file 1: Fig. S6). For each translocation experiment, transepithelial electrical resistance (TEER) was measured before and after AuNP exposure. TEER values were slightly decreased only after Au-4-COONa NP treatment of the BeWo cell layer and the co-culture (Additional file 1: Fig. S5).

### Determination of AuNP distribution in the ex vivo placenta perfusion model

**Fig. 5:**
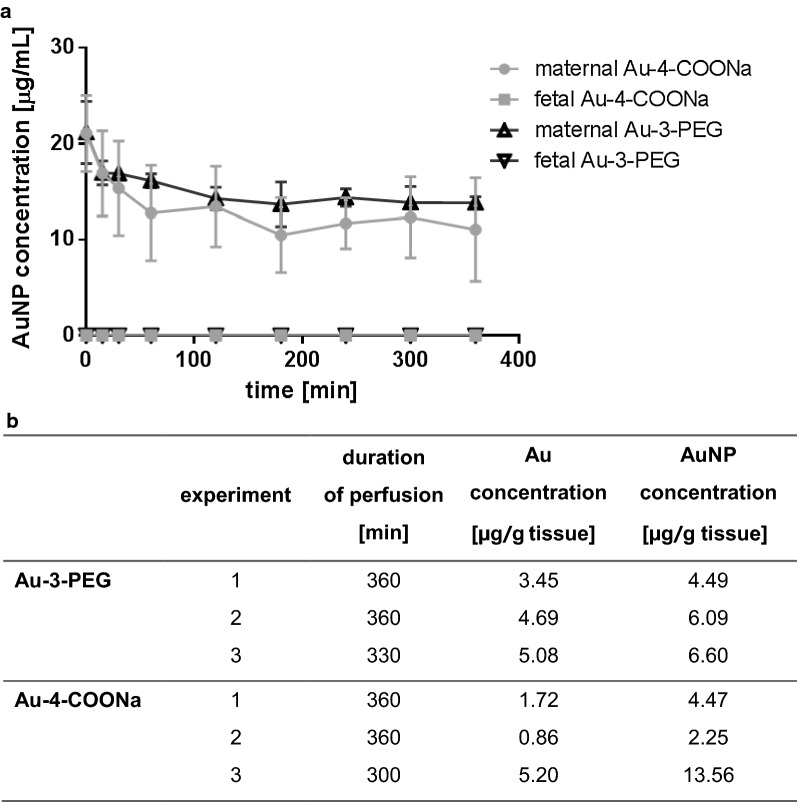
Perfusion kinetics and placental tissue accumulation of Au-3-PEG and Au-4-COONa NPs determined by SF-ICP-MS. **a** Maternal and fetal AuNP concentration over time (data presented as mean ± SD). AuNP concentrations were calculated from the Au concentrations measured with SF-ICP-MS using the relative Au content of the NPs determined with ICP-OES (see Table 1). **b** Placental tissue accumulation of Au-3-PEG and Au-4-COONa NPs after 5–6 h of perfusion, shown as Au and AuNP concentration (calculated as indicated before)

**Fig. 6:**
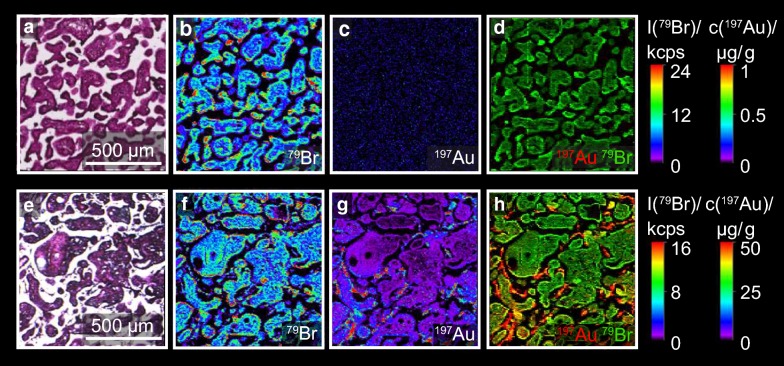
Distribution of Au-4-PEG NPs in placental tissue after 6 h of perfusion. Placental tissue was perfused with 25 μg/mL Au-3-PEG NPs for 6 h. **a**, **e** H&E staining of placental tissue before (**a**) and after perfusion (**e**). **b**, **f** Elemental distribution of ^79^Br to visualize tissue structure. **c**, **g** ^197^Au was measured to localize Au. **d**, **h** Overlay of ^79^Br and ^197^Au to co-localize Au signals in placental tissue. Elemental distribution map of ^79^Br and ^197^Au is represented in signal intensities and Au concentrations (black: minimum, red: maximum), respectively. Images were obtained from one independent experiment

Placental translocation of Au-3-PEG and Au-4-COONa NPs was determined using the ex vivo perfusion of term placental tissue. Here, a concentration of 25 μg/mL AuNPs was used for both, the PEGylated and carboxylated AuNPs. As shown in Fig. 5a, similar translocation kinetics of Au-3-PEG and Au-4-COONa NPs were observed during 6 h of perfusion. The AuNP concentration decreased from 25 μg/mL to 13.76 μg/mL and 10.45 μg/mL of Au-3-PEG and Au-4-COONa NPs, respectively. Both, Au-3-PEG NPs and Au-4-COONa NPs, were found to accumulate to a similar amount in placental tissue after 5–6 h of perfusion (4–7 μg/g tissue for Au-3-PEG NPs vs. 2–14 μg/g tissue for Au-4-COONa NPs). No or very low translocation to the fetal side was observed for Au-4-COONa and Au-3-PEG NPs (0 and 0.0031 μg/mL after 6 h; n = 2), respectively. To assess adsorption of the AuNPs to the perfusion device, control perfusions were done in the absence of placental tissue (Additional file 1: Fig. S7). These experiments demonstrated that Au-3-PEG NPs stayed in suspension over 6 h of perfusion, whereas the Au-4-COONa NP concentration dropped after around 3 h of perfusion, indicating sedimentation or binding of these AuNPs to the tubes and/or system components. Therefore, LA-ICP-MS analysis was only performed from placental tissue before and after 6 h of perfusion with Au-3-PEG NPs (Fig. 6). Around 7.9 µg Au/g tissue was found within ^79^Br borders after 6 h of perfusion. Au-3-PEG NPs were found in the outer layer of the placental tissue, namely the ST, but were also able to penetrate into the placental tissue. Few detached ST fragments with high Au signals were observed. However, such CK7-positive fragments were already present before perfusion with AuNPs or after 6 h perfusion with PM only (Additional file 1: Fig. S8).

## Discussion

### Choice of AuNPs and placenta models

Small 3–4 nm AuNPs were selected for this study since placental transfer appears to inversely correlate with particle size [6] and considerably increased translocation rates have been reported for AuNPs smaller than approx. 10 nm at the alveolar epithelial tissue barrier [18]. To exploit the impact of different surface modifications on placental translocation, PEGylated and carboxylated AuNPs were included. Since PEGylation is known to reduce NP-cell and NP–protein interactions, less accumulation in placental tissue was expected compared to carboxylated AuNPs [29].

Relatively high concentrations of 25–50 µg/mL AuNPs were chosen, which are representative of a medically relevant exposure scenario where blood concentrations up to 100 μg/mL are conceivable [30]. Environmental exposure of pregnant women to AuNPs released from consumer products or nanomedical applications to the environment is currently expected to be very low [31] and outer tissue barriers such as the lung, skin or intestine have been shown to greatly limit NP transfer to the systemic circulation [18, 32].

The ex vivo perfusion of human term placental tissue is considered as gold standard for human placental translocation studies since it retains the structural complexity and recapitulates the dynamic environment of the in vivo tissue. However, perfusion studies are limited to a few hours, have a low success rate (approx. 30%) and provide limited mechanistic insights [9, 26]. Consequently, in vitro transfer models based on microporous supports are frequently used as a complementary approach with increased throughput capability and the possibility to address permeability across individual cell layers [25, 33–35]. Here, we applied our recently developed human placental co-culture model to investigate translocation across the trophoblast layer (BeWo cells), endothelial layer (HPEC) or the co-culture [25]. It has been shown that the translocation rates of small molecules obtained using these cell- and tissue-based models are in good correlation with each other and that ex vivo translocation rates predict relatively well the in vivo situation [36, 37]. However, the predictive value of these models for NP translocation studies remains to be proven [6, 34].

### Colloidal stability of AuNPs in biological media

If NPs are added to a biological matrix, constituents like proteins and lipids can adhere to the particles, forming a so-called bio- or protein corona [38–40]. This can lead to an agglomeration of the particles followed by sedimentation and thereby alter their interaction with cells [41]. Since the formation of a corona is highly dependent on the properties of the NP and the composition of the media, we examined potential agglomeration and sedimentation of both AuNP types in H_2_O, EM (in vitro) and PM (ex vivo) by visual inspection as well as SAXS and DLS measurements. In water, both AuNPs stayed in suspension after 24 h incubation at 37 °C without a substantial size change. High colloidal stability was also observed for Au-3-PEG NPs dispersed in PM and EM, demonstrating the ability of PEG to restrict protein NP interactions [29]. In contrast, strong agglomeration was observed for Au-4-COONa NPs in PM and EM. However, limitations of the SAXS and DLS approaches hamper the identification of exact particle sizes. In SAXS, the observation window is weighted towards particles below its detection limit (~ 100 nm) while in DLS (due to the larger wavelength of lasers with respect to X-ray), the signal is more weighted by larger particles. Therefore, SAXS analysis can indicate precipitation of agglomerated particles due to intensity loss in the supernatants, while DLS measurements can detect larger NP agglomerates but fails in giving exact particle sizes for polydisperse samples [42]. A possible explanation for the agglomeration of Au-4-COONa but not Au-3-PEG NPs may be a partial loss of the stabilizing carboxyl-ligand since thiol-ligands can be released from AuNPs in particular in in vivo settings. In a previous work it has been shown that AuNPs functionalized with thiol-ending PEG were most stable in aqueous solutions compared to other thiol-compounds [43]. Although colloidal stability of the AuNPs was investigated in the relevant biological media and for the same incubation times as in the experimental setup, we cannot exclude that the agglomeration behavior of the AuNPs may be slightly different under experimental conditions in the presence of cells/tissue or dynamic exposure conditions. Indeed, we observed that almost all Au-4-COONa NPs adhered to the ex vivo perfusion device in an empty perfusion already after 3 h of perfusion, whereas a considerable amount of NPs remained in the maternal circulation for up to 6 h in the presence of placental tissue (Fig. 5a and Additional file 1: Fig. S7). Proteins released from the placental tissue and/or the slightly different pressure conditions may account for the observed differences.

### Placental translocation of AuNPs in vitro

Translocation studies revealed that Au-3-PEG and Au-4-COONa NPs could pass the in vitro co-culture placental barrier in low amounts after 24 h of exposure (0.6% and 0.1% of the ID, respectively). Although these values were low, the determined Au concentrations were clearly above the detection limit of the ICP-MS analytics (0.014% of the ID for both AuNPs). Both, the trophoblast and endothelial cell layer exhibited a high barrier capacity for Au-4-COONa NPs while for Au-3-PEG NPs, the endothelial cell layer showed a lower retention potential than the trophoblast layer. Although both AuNPs were not cytotoxic to BeWo cells at concentrations of 50 µg/mL for up to 48 h of exposure, a small decrease in the TEER was noted after translocation studies with Au-4-COONa NPs for 24 h indicating a potential loss in barrier integrity. Whether this slight decrease in TEER value is sufficient to allow penetration of small molecules or even NPs as well as the long-term consequences of carboxylated AuNP accumulation on barrier integrity need to be carefully addressed in future studies. No impact on TEER was observed after treatment with Au-3-PEG NPs, indicating that at least in this case, particle transfer across an intact co-culture barrier was possible.

Interestingly, a slightly higher transfer was found for Au-3-PEG NPs despite the fact that deposited doses were probably lower compared to Au-4-COONa NPs due to their strong agglomeration in the culture medium. Indeed, substantial amounts of Au-4-COONa NPs were detected in the membrane fraction by ICP-MS and predominantly localized to the trophoblast layer as evidenced by LA-ICP-MS. Since conventional ICP-MS and LA-ICP-MS cannot discriminate between ionic and particulate Au, TEM analysis was employed to confirm cellular uptake of Au-4-COONa NPs. Small nanosized but no microsized agglomerates of Au-4-COONa NPs were observed in membrane-bound vesicles. Therefore, agglomeration may at least partially account for the reduced translocation compared to well-dispersed Au-3-PEG NPs. No particles or agglomerates were observed in TEM micrographs of BeWo cells exposed to Au-3-PEG NPs possibly due to the very low amounts of internalized Au-3-PEG NPs as evidenced by SF-ICP-MS and LA-ICP-MS.

This differential uptake and accumulation behavior of Au-3-PEG and Au-4-COONa NPs observed in the co-culture transfer model was highly similar to the findings described in our previous study using 3D placental co-culture MTs [21]. Au-4-COONa NPs were internalized in higher amounts than Au-3-PEG NPs and accumulated mostly in the outer BeWo cell layer of the MT. Furthermore, studies with placental co-culture MTs provided additional information on the ability of AuNPs to penetrate into the fibroblastic core after crossing the trophoblast barrier, which is more difficult to obtain in the in vitro co-culture transfer model due to the presence of an artificial microporous membrane and challenges to detect the NPs in the delicate endothelial cell layer. In placental MTs it has been shown that internalized Au-3-PEG NPs crossed the BeWo layer and penetrated into the fibroblastic core of the MTs [21], whereas in the in vitro placental transfer model, internalization of Au-3-PEG NPs by endothelial cells was unclear due to the low resolution limit of the LA-ICP-MS technique (2 μm). Therefore, the combination of these two in vitro placental models can deliver complementary insights into NP uptake and translocation at the placental barrier.

### Placental translocation of AuNPs ex vivo

Although, the outcomes obtained with the placental MT and the co-culture transfer model are fairly consistent, these in vitro approaches have some limitations. In both models, the BeWo cell line was used to mimic the ST barrier. While the ST forms a syncytium in vivo, BeWo cells do not fuse spontaneously and consist mainly of undifferentiated cytotrophoblasts with a few syncytialized cells. A further shortcoming is the lack of a physiological microenvironment, since both in vitro studies were conducted under static conditions. However, a dynamic model could be crucial for reliable NP studies [6] since NPs exhibit unique agglomeration and sedimentation behavior in biological media that can have a significant impact on the bioavailable dose [22, 23]. Moreover, flow conditions may further affect cellular doses, transport kinetics of NPs as well as cell performance as shown in recent studies, where dynamic models were capable of producing more predictive doses and accurate outcomes compared to static systems [24, 44]. Therefore, we additionally investigated translocation kinetics of Au-3-PEG and Au-4-COONa NPs using the dynamic ex vivo placenta perfusion model. Similar to the in vitro studies, AuNP translocation was limited, and a slightly higher transfer was observed for Au-3-PEG (0.01% of ID; detection limit: 0.0052% of ID) compared to Au-4-COONa NPs, which did not cross the placental barrier after 5–6 h of perfusion (detection limit: 0.0085% of ID). However, due to the considerable agglomeration of Au-4-COONa NPs in PM and non-specific adherence to the perfusion system, the available cellular doses are likely much lower than for perfusions with Au-3-PEG NPs or experiments in the static in vitro models. Dosimetry issues may also explain why AuNP tissue concentrations were similar in ex vivo perfusion studies while much higher cellular uptake was detected for Au-4-COONa NPs in the static in vitro models. According to LA-ICP-MS analysis, Au-3-PEG NPs penetrated and distributed throughout the placental tissue, which has also been described in 3D placental MTs [21]. In few cases, ST fragments were observed that appeared to accumulate slightly larger amounts of Au-3-PEG NPs than the intact ST layer, possibly due to a higher available surface area of these fragments. ST fragments were already present before the perfusion with NPs, excluding major adverse effects of AuNPs on ST barrier integrity. Moreover, no leakage was observed during 6 h of perfusion, which further supports placental barrier tightness. It is possible that these fragments were already present in the placental tissue after delivery or they may represent an artefact from sample processing for histological analysis.

Overall, in vitro and ex vivo placental transfer models delivered fairly consistent and complementary results on the relative uptake and translocation of Au-3-PEG and Au-4-COONa NPs when accounting for differences in particle stability and non-specific adherence to the test system. Nevertheless, a direct comparison is challenging due to differences in deposited AuNP concentrations, culture media and/or duration of exposure. For stable NPs such as Au-3-PEG NPs, low placental translocation in vitro and ex vivo correlates well with findings from placental translocation studies in rodents [14, 15].

## Conclusions

In this study, we provide the first evidence for the translocation of small 3–4 nm AuNPs across the human placental barrier both in vitro and ex vivo. Although only very low percentages of the applied doses did cross the placental barrier, this still represents a considerable number of individual particles. Since the developing fetus is particularly vulnerable to toxic substances, potential embryo-fetotoxic and teratogenic effects of AuNPs should be carefully investigated. However, as our models represent the final stages of pregnancy, it remains to be shown if small AuNPs can also cross the early human placental barrier. So far, it is unclear if the embryo is better or less protected against effects of NP exposure during early versus later stages of pregnancy (as reviewed by Juch et al. [45]). In general, it is assumed that NP translocation across the placental barrier is lower during early pregnancy due to the increased thickness of the placental barrier, but, substantial accumulation and persistence of AuNPs in placental tissue could interfere with proper placental development and function. For instance, we observed a decrease in TEER after exposure to carboxylated AuNPs suggesting that barrier integrity may be compromised. In addition, studies in pregnant rodents provide increasing evidence that NPs can damage the placental barrier or alter the release of placental mediators which may at least partially account for the observed fetotoxic effects [46–50].

To support the safe and sustainable use of AuNPs, we should seek to understand if AuNPs impact on human placental function thereby causing a hostile gestational environment for fetal growth, development and maturation. Finally, to more clearly elucidate the impact of different NP properties and modifications on placental uptake and translocation, NPs with high stability in biological media are needed to exclude a superimposed size-dependent effect.

A prerequisite to achieve novel insights on NP-placenta interactions is a thorough NP characterization as well as the use of predictive human placenta models. In this regard, our study suggests that results on AuNP placental tissue accumulation as well as placental translocation obtained with human in vitro and ex vivo placenta models correlate well with each other and with rodent studies. However, careful assessment of NP stability and non-specific interference with the test system is indispensable for the proper interpretation of the results and for comparison between different models.

## Additional file

**Additional file 1: Fig S1.** Colloidal stability of Au-3-PEG and Au-4-COONa NPs in PM and ultrapure water. **Fig S2.** Size distribution of Au-4-COONa and Au-3-PEG NPs in ultrapure water, EM and PM measured by DLS at 37 °C over time. **Fig S3.** Light microscopic images of Au-4-COONa and Au-3-PEG NP suspensions after 6 h and 24 h incubation at 37 °C/5% CO_2_ under static conditions. **Fig S4.** Effect of Au-3-PEG and Au-4-COONa NPs on BeWo viability. **Fig S5.** Transepithelial electrical resistance (TEER) before and after 24 h of AuNP treatment. **Fig S6.** TEM micrographs of BeWo cells after exposure to AuNP for 24 h. **Fig S7.** Adsorption of Au-4-COONa and Au-3-PEG NPs in the ex vivo perfusion device. **Fig S8.** Cytokeratin 7 (CK7) staining of human placental tissue before and after 6 h of ex vivo perfusion (without NPs).
