# Supplementary material for: Gold nanoparticle distribution in advanced in vitro and ex vivo human placental barrier models
Source: J Nanobiotechnology. 2018 Oct 11;16:79. doi: 10.1186/s12951-018-0406-6 (PMC6180500; doi:10.1186/s12951-018-0406-6)
Supplement: Supplementary file 1 — Additional file 1: Fig S1. Colloidal stability of Au-3-PEG and Au-4-COONa NPs in PM and ultrapure water. Fig S2. Size distribution of Au-4-COONa and Au-3-PEG NPs in ultrapure water, EM and PM measured by DLS at 37 °C over time. Fig S3. Light microscopic images of Au-4-COONa and Au-3-PEG NP suspensions after 6 h and 24 h incubation at 37 °C/5% CO2 under static conditions. Fig S4. Effect of Au-3-PEG and Au-4-COONa NPs on BeWo viability. Fig S5. Transepithelial electrical resistance (TEER) before and after 24 h of AuNP treatment. Fig S6. TEM micrographs of BeWo cells after exposure to AuNP for 24 h. Fig S7. Adsorption of Au-4-COONa and Au-3-PEG NPs in the ex vivo perfusion device. Fig S8. Cytokeratin 7 (CK7) staining of human placental tissue before and after 6 h of ex vivo perfusion (without NPs). [file 12951_2018_406_MOESM1_ESM.docx]

**Additional information**

**Gold nanoparticle distribution in advanced *in vitro* and *ex vivo* human placental barrier models**

Leonie Aengenheister^1^, Dörthe Dietrich^2^, Amin Sadeghpour^3^, Pius Manser^4^, Liliane Diener^5^, Adrian Wichser^6^, Uwe Karst^7^, Peter Wick^8^, Tina Buerki-Thurnherr^9,*^

^1^ Empa, Particles-Biology Interactions, Swiss Federal Laboratories for Materials Science and Technology, Lerchenfeldstrasse 5, 9014 St. Gallen, Switzerland; leonie.aengenheister@empa.ch

^2^ Institute of Inorganic & Analytical Chemistry, Westfälische Wilhelms-Universität Münster, Corrensstraße 28/30, 48149 Münster, Germany; doerthe.dietrich@uni-muenster.de

^3^ Empa, Center for X-ray analytics, Swiss Federal Laboratories for Materials Science and Technology, Lerchenfeldstrasse 5, 9014 St. Gallen, Switzerland; amin.sadeghpour@empa.ch

^4^ Empa, Particles-Biology Interactions, Swiss Federal Laboratories for Materials Science and Technology, Lerchenfeldstrasse 5, 9014 St. Gallen, Switzerland; pius.manser@empa.ch

^5^ Empa, Particles-Biology Interactions, Swiss Federal Laboratories for Materials Science and Technology, Lerchenfeldstrasse 5, 9014 St. Gallen, Switzerland; liliane.diener@empa.ch

^6^ Empa, Laboratory for Advanced Analytical Technologies, Swiss Federal Laboratories for Materials Science and Technology, Ueberlandstrasse 129, 8600 Duebendorf, Switzerland; adrian.wichser@empa.ch

^7^ Institute of Inorganic & Analytical Chemistry, Westfälische Wilhelms-Universität Münster, Corrensstraße 28/30, 48149 Münster, Germany; uk@uni-muenster.de

^8^ Empa, Particles-Biology Interactions, Swiss Federal Laboratories for Materials Science and Technology, Lerchenfeldstrasse 5, 9014 St. Gallen, Switzerland; peter.wick@empa.ch

^9^ Empa, Particles-Biology Interactions, Swiss Federal Laboratories for Materials Science and Technology, Lerchenfeldstrasse 5, 9014 St. Gallen, Switzerland; tina.buerki@empa.ch

*corresponding author: tina.buerki@empa.ch

*MTS viability assay*

The influence of Au-4-COONa and Au-3-PEG NPs on cell viability was assessed using the MTS assay. BeWo cells were seeded in a 96-well plate (1 x 10^4^ cells per well) for 24 h and subsequently treated with different concentrations of AuNPs. As negative control, cells without treatment were used and as positive control 1 mM CdSO_4_ was applied. After incubation (6 h, 24 h, 48 h) at 37 °C and 5% CO_2_, the MTS assay (CellTiter96® AQueous One Solution Cell Proliferation Assay, Promega, Dübendorf, Switzerland) was performed according to the manufacturer’s instructions. Optical density was measured at 490 nm with a microplate reader (Mithras2 LB 943, Berthold Technologies GmbH, Zug, Switzerland). OD values were blank-corrected and normalized to untreated controls. Potential interference of the NPs with the MTS assay was excluded beforehand.

*Transepithelial electrical resistance (TEER)*

The TEER was measured before the *in vitro* translocation studies to confirm a proper barrier formation of the monolayers and co-culture as well as afterwards to determine the influence of the AuNP treatment on barrier integrity. TEER was determined on the collagen-coated inserts in the presence or absence of cells using a chopstick electrode (STX3, World Precision Instruments Inc., Sarasota, USA). TEER values for the cell layer were obtained by subtracting the intrinsic resistance (blank insert membrane) from the total resistance (insert membrane with cells) and were corrected for the surface area (Ω cm^2^).

*Immunohistochemical (IHC) staining for cytokeratin 7 (CK7)*

CK7 stainings were perfomed on 5 µm sections of paraffin embedded placental tissue taken before and after a control *ex vivo* perfusion with PM only (without NPs). Murine colon was used as a negative control. After deparaffinization, antigen retrieval was performed by incubating the sections in citrate buffer (pH 6.0) for 80 min at 95 °C. Sections were washed in PBS and endogenous peroxidase was quenched with 0.5% H_2_O_2_ in PBS for 30 min at RT. Between the main steps (H_2_O_2_, block, primary and secondary antibody), sections were washed 3 times with PBS for 10 min at RT. After blocking with 5% goat serum in 0.3% PBS-T (PBS with 0.3% Tween-20) at RT for 1 h, sections were incubated with mouse-anti-CK7 antibody (OV-TL 12/30, Dako, Agilent, Basel, Switzerland; 1:50) diluted in 0.5% bovine serum albumin (BSA) in PBS at 4 °C overnight, followed by incubation with goat-anti-mouse IgG-HRP (Jackson ImmunoResearch Laboratories Inc., West Grove, PA, USA; 1:100) in 0.5% BSA in PBS at RT for 1 h. The staining was developed using freshly prepared DAB solution (Dako Liquid DAB+ Substrate, Agilent, Basel, Switzerland) for 3-5 min and sections were washed once with PBS and counterstained with hematoxylin. Finally, sections were dehydrated and embedded using Roti^®^-Histokitt II (Carl Roth **GmbH + Co. KG, Karlsruhe, Germany**). Light microscopic images were taken with a Leica DFC450C camera coupled to a Leica DM4000 B LED microscope (Leica Microsystems Ltd., Heerbrugg, Switzerland).

*Adsorption of AuNPs in the ex vivo perfusion device*

To determine a potential loss of freely diffusing AuNPs during the *ex vivo* perfusion, a perfusion in the closed maternal compartment was performed. Therefore, the cannulas of the maternal device were put into the cylinder of the maternal compartment instead of being inserted into the placental tissue. Same AuNP concentration (25 µg/mL), but lower volume (30 mL) was used as in the *ex vivo* perfusion studies. At each time point (0, 0.25, 0.5, 1, 2, 3, 4, 5 and 6 h) 2 mL samples were taken and stored at -20 °C for further SF-ICP-MS analysis. 250 µL aliquots of each sample were digested in 0.6 mL concentrated nitric acid and 1.8 mL hydrochlorid acid using a microwave and were further diluted with ultrapure water as described for the samples from the NP perfusion studies. Rhenium was added as internal standard and the Au content was quantified via an external calibration ranging from 0 – 50 µg/L (isotope ^197^Au, low resolution).

*Statistics*

The experimental data is presented as mean ± standard deviation (SD). To find statistical difference in cell viability after different treatment conditions (MTS assay), each treatment condition was compared to the untreated control using an unpaired student’s t-test. Statistical significance was obtained when p< 0.05 (*).


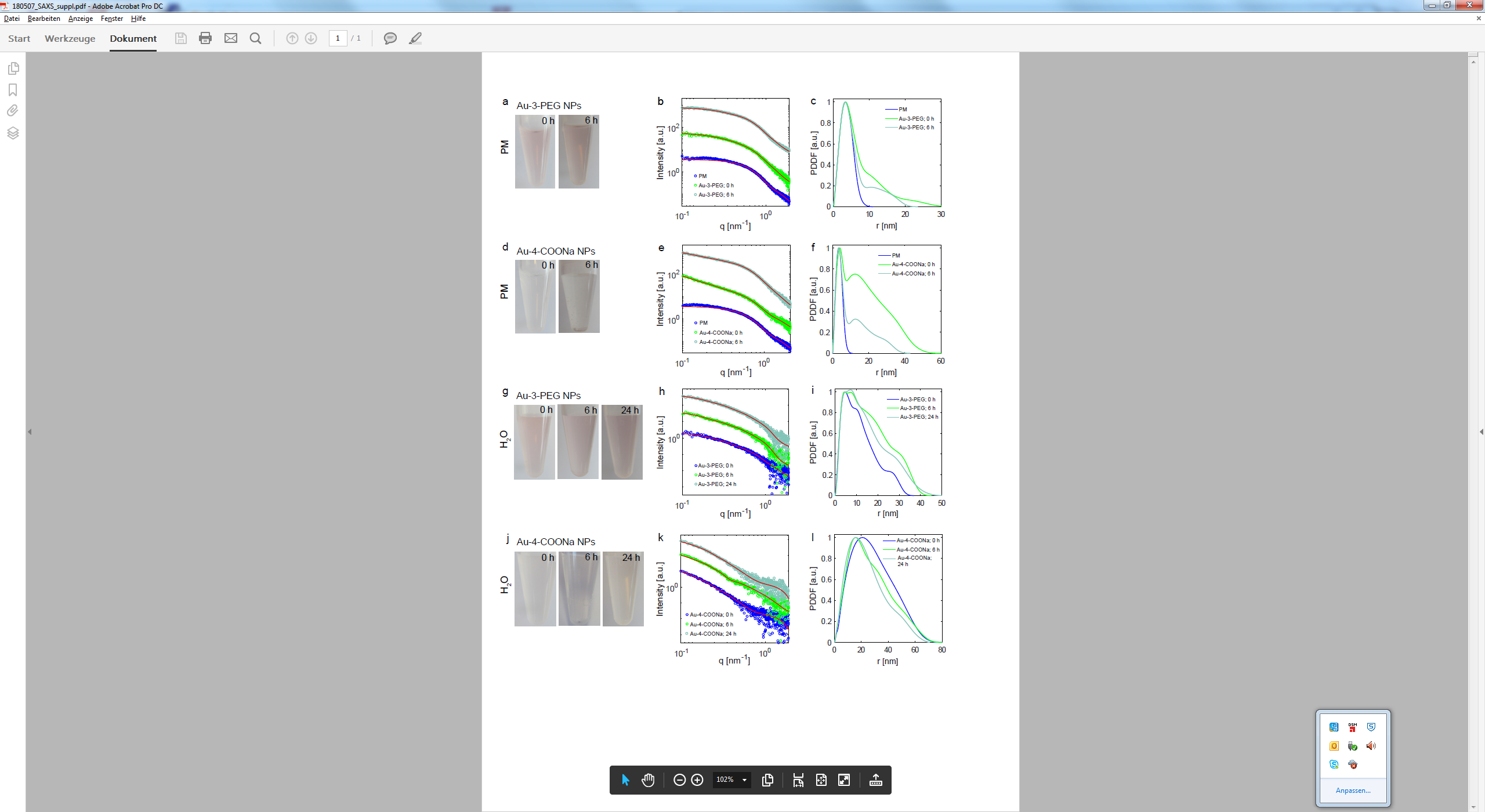


Fig. S1: Colloidal stability of Au-3-PEG (a-c; g-i) and Au-4-COONa (d-f; j-l) NPs in PM and ultrapure water. (a, d, g, j) Au-3-PEG and Au-4-COONa NP suspensions in PM (25 μg/mL AuNP each) and water (19.2 μg/mL Au each) after 0, 6 and 24 h of static incubation (37 °C/5% CO_2_) in the absence of cells. No 24 h timepoint was included for experiments with PM since *ex vivo* perfusions studies were limited to 6 h. (b, e, h, k) Scattering intensity as function of scattering vector q and (c, f, i, l) pair distance distribution function (PDDF) of the PEGylated and carboxylated AuNPs in PM and water.


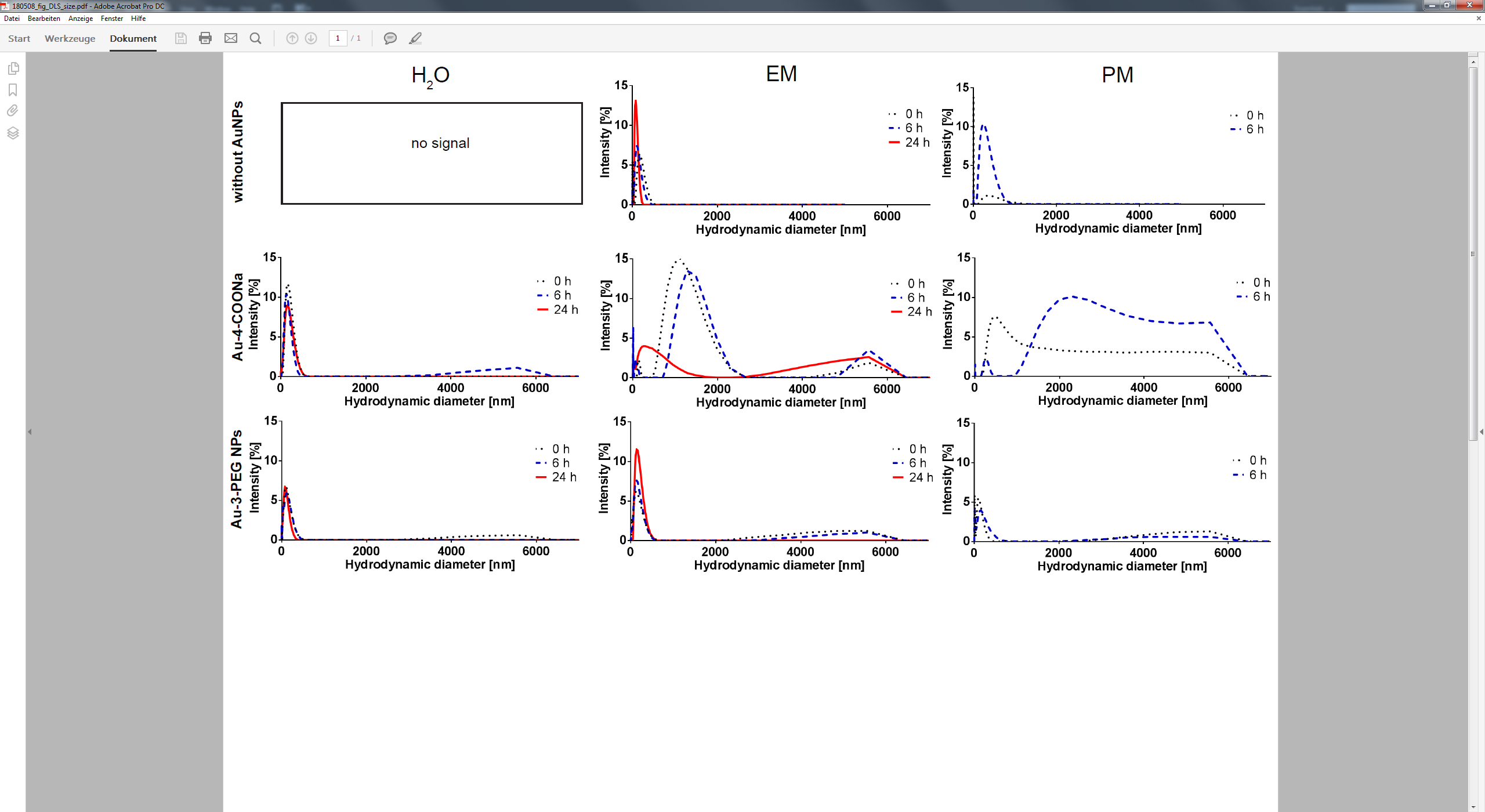


Fig. S2: Size distribution of Au-4-COONa and Au-3-PEG NPs in ultrapure water, EM and PM measured by DLS at 37 °C over time. The hydrodynamic diameter of the AuNPs was measured in 100 µg/mL AuNP suspensions in EM, PM and ultrapure H_2_O at 37 °C for 0, 6 and 24 h, respectively.


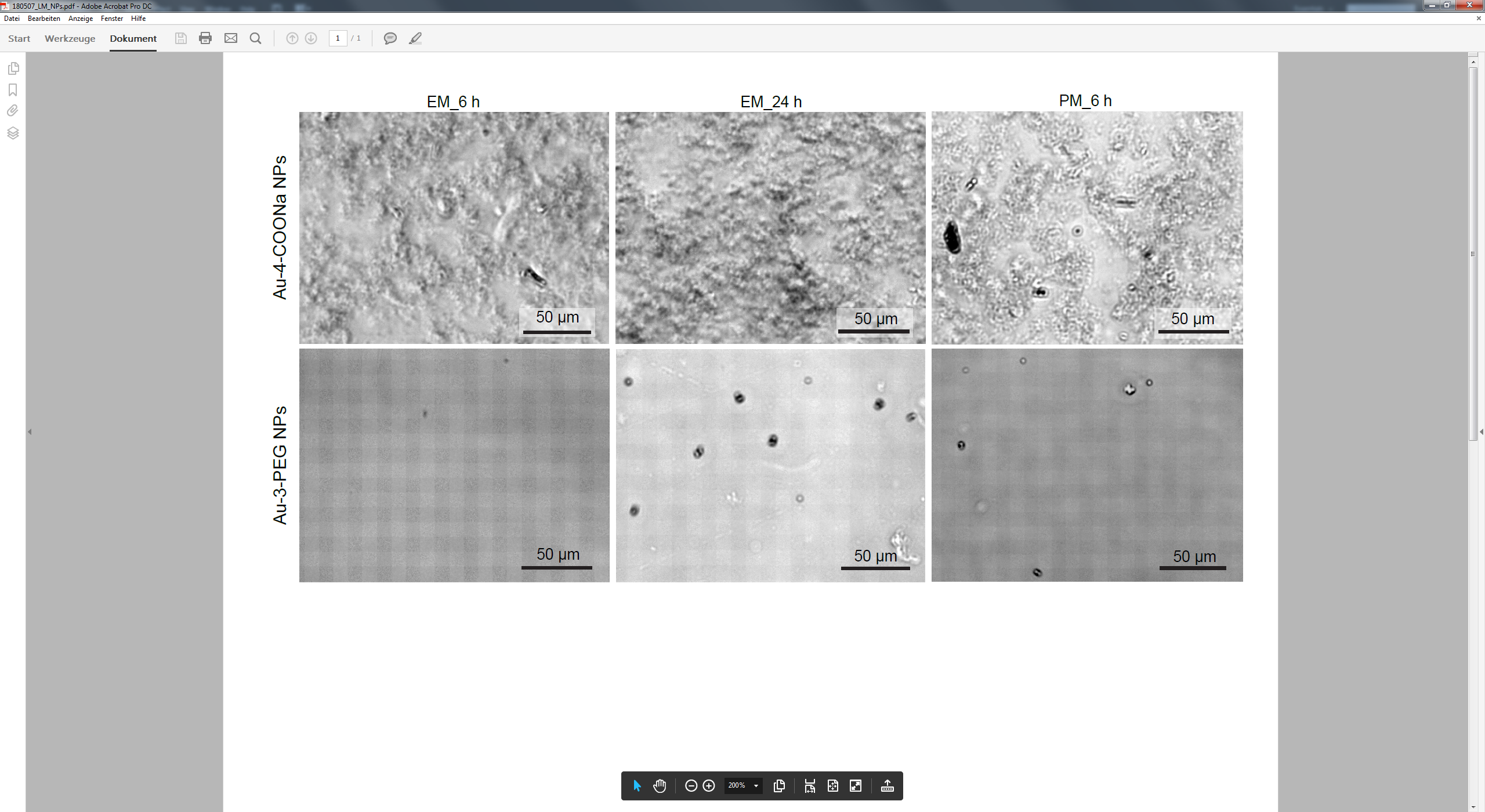


Fig. S3: Light microscopic images of Au-4-COONa and Au-3-PEG NP suspensions after 6 h and 24 h incubation at 37 °C/ 5% CO_2_ under static conditions. AuNP suspensions (50 µg/mL of Au-4-COONa in EM; all other suspensions 25 µg/mL of the respective AuNP) were incubated in 24 well plates in the absence of cells (images were taken at the bottom of the wells).


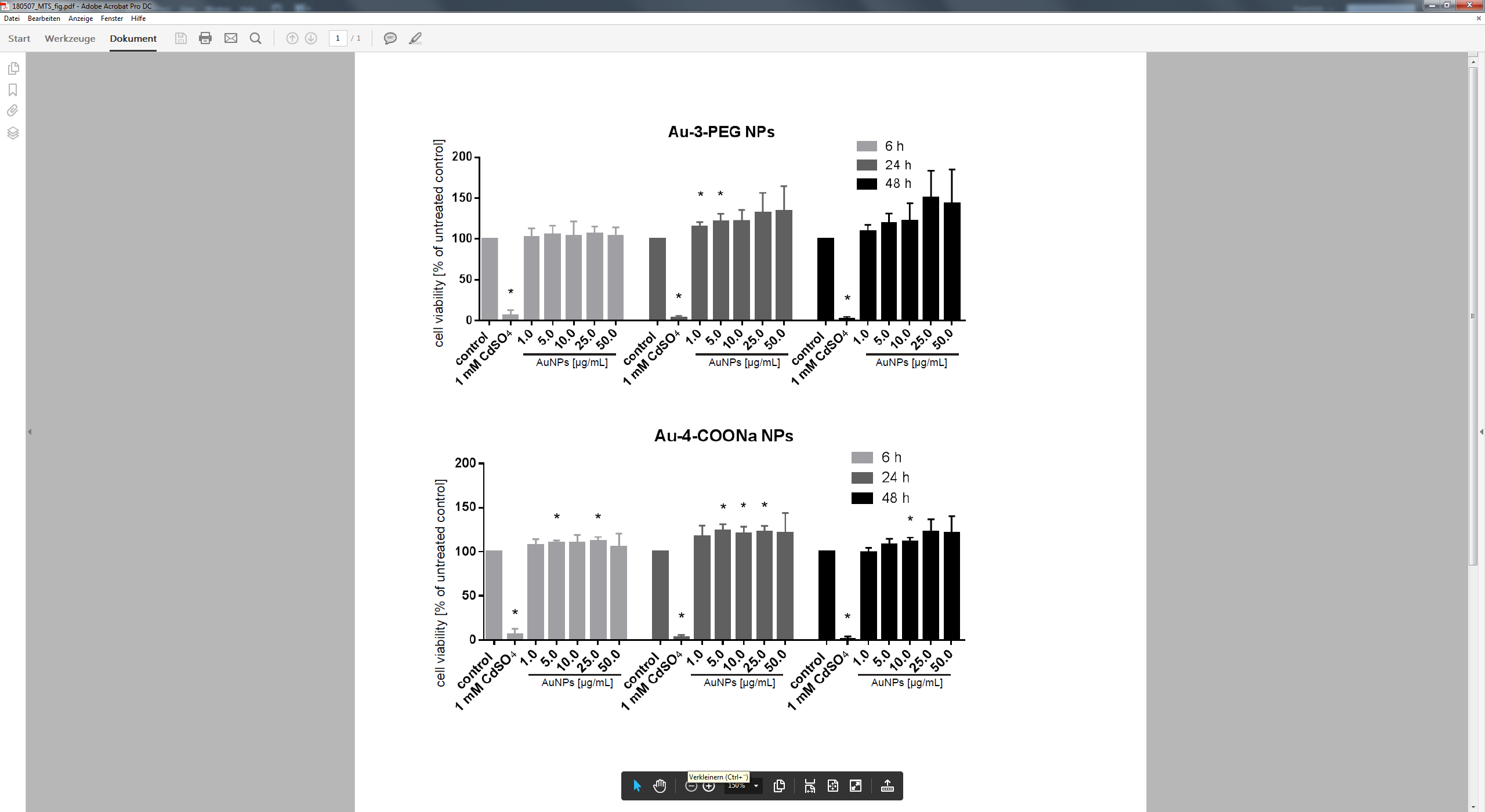


Fig. S4: Effect of Au-3-PEG and Au-4-COONa NPs on BeWo viability. Cell viability was assessed after treatment with 0.01-50 µg/mL AuNPs for 6, 24 and 48 h using the MTS assay. 1 mM CdSO_4_ was applied as positive control. Data represent the mean ± SD of three biologically independent experiments with three technical replicates each. Student’s t-test was performed to find significance between the untreated control group (EM) and different treatment conditions (*p<0.05 was considered significant).


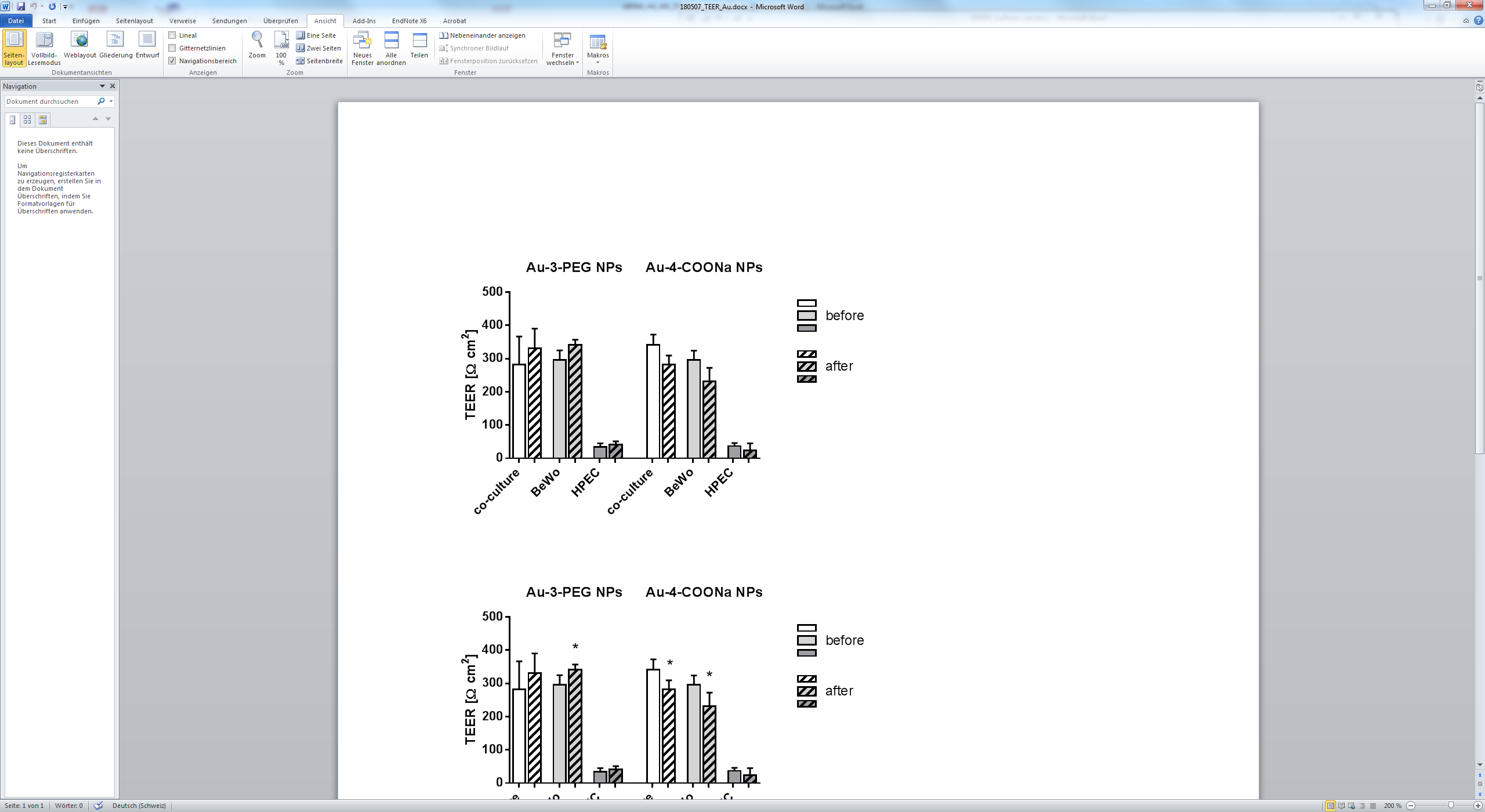


Fig. S5: Transepithelial electrical resistance (TEER) before and after 24 h of AuNP treatment. Data is represented as mean ±SD


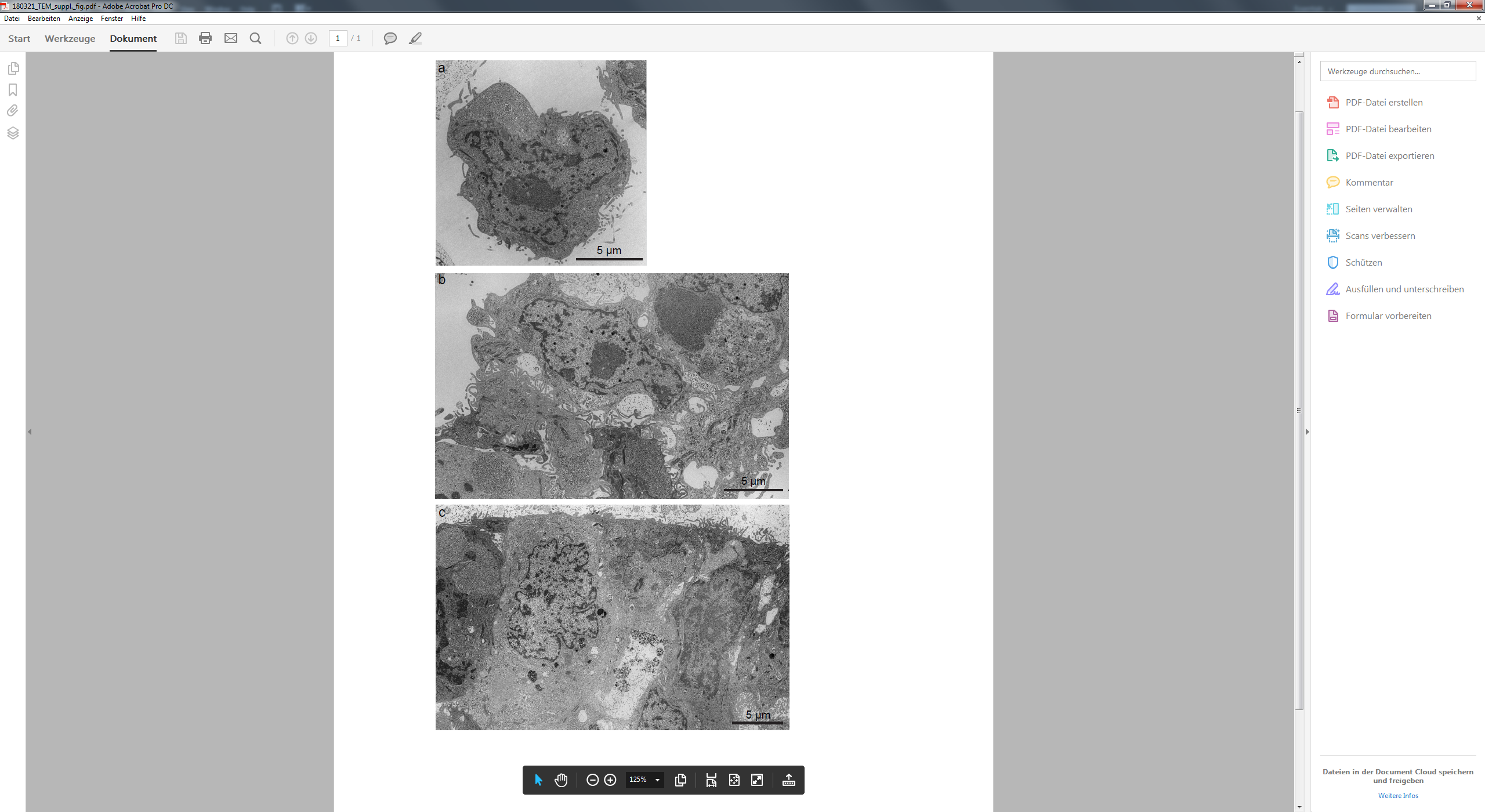


Fig. S6: TEM micrographs of BeWo cells after exposure to AuNP for 24 h. Cells were treated with EM only (a), 25 µg/mL Au-3-PEG (b) or 50 µg/mL Au-4-COONa NP (c; both AuNP concentrations corresponding to 19.2 µg/mL Au).


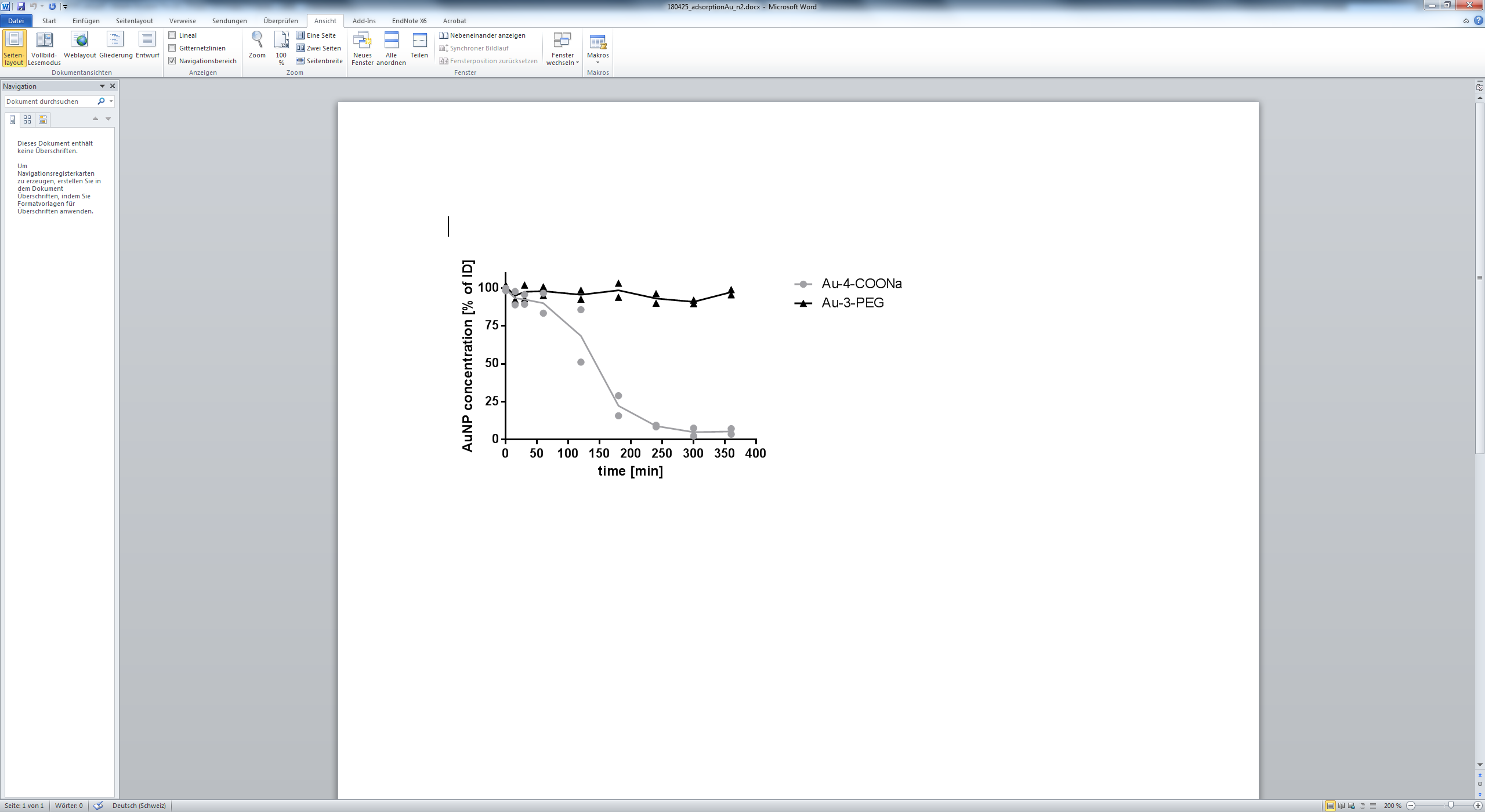


Fig. S7: Adsorption of Au-4-COONa and Au-3-PEG NPs in the *ex vivo* perfusion device. The graph shows the available AuNPs in the perfusion medium over time, when added to the maternal compartment of the *ex vivo* perfusion device (without placental tissue). 25 µg/mL of each NP was initially applied. The same parameters (e.g. temperature, mixing and perfusion rate) were used as for the *ex vivo* perfusion of placental tissue (n=2; values of each replicate are shown; line is indicating the mean value).


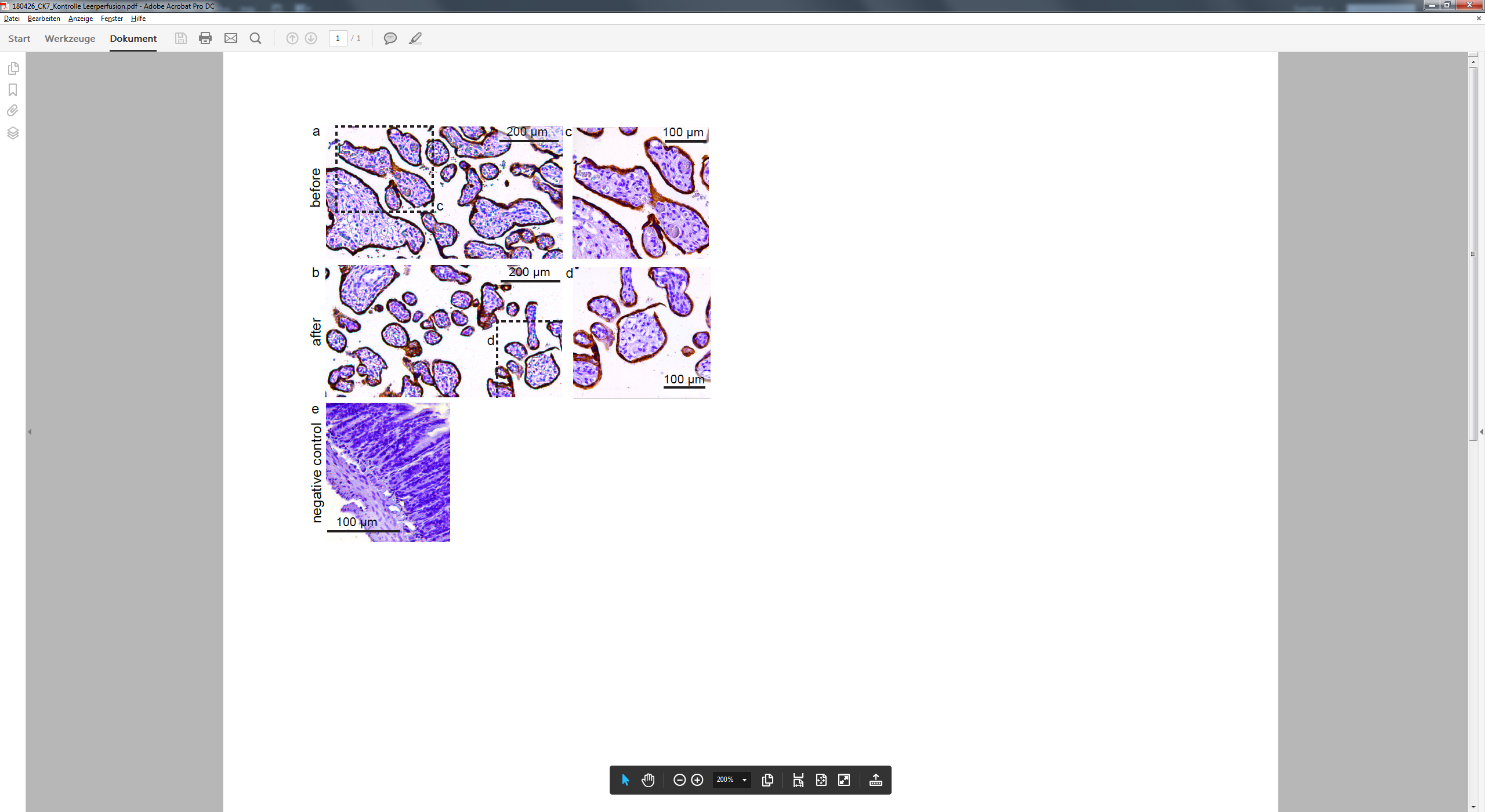


Fig. S8: Cytokeratin 7 (CK7) staining of human placental tissue before (a, c) and after 6 h of *ex vivo* perfusion (b, d) (without NPs). Paraffin sections (5 µm thick) were stained for CK7 (brown) to detect the syncytiotrophoblast and counterstained with hematoxylin (blue) for overall cell structure. The dashed line indicates the area of each magnification on the left (c, d). Murine colon was used as CK7-negative control (e).
